# Cancer immunotherapy with PI3K and PD-1 dual-blockade via optimal modulation of T cell activation signal

**DOI:** 10.1136/jitc-2020-002279

**Published:** 2021-08-20

**Authors:** Sho Isoyama, Shigeyuki Mori, Daisuke Sugiyama, Yasuhiro Kojima, Yasuko Tada, Kohei Shitara, Kunihiko Hinohara, Shingo Dan, Hiroyoshi Nishikawa

**Affiliations:** 1 Division of Cancer Immunology, Research Institute/Exploratory Oncology Research and Clinical Trial Center (EPOC), National Cancer Center, Tokyo/Kashiwa, Japan; 2 Division of Molecular Pharmacology, Cancer Chemotherapy Center, Japanese Foundation for Cancer Research, Tokyo, Japan; 3 R&D Center, Zenyaku Kogyo Co Ltd, Tokyo, Japan; 4 Department of Immunology, Nagoya University Graduate School of Medicine, Nagoya, Japan; 5 Department of Gastroenterology and Gastrointestinal Oncology, National Cancer Center Hospital East, Kashiwa, Japan

**Keywords:** immunotherapy, immunologic memory, drug therapy, combination, tumor microenvironment

## Abstract

**Background:**

Immune checkpoint blockade (ICB) induces durable clinical responses in patients with various types of cancer. However, its limited clinical efficacy requires the development of better approaches. In addition to immune checkpoint molecules, tumor-infiltrating immunosuppressive cells including regulatory T cells (Tregs) play crucial roles in the immune suppressive tumor microenvironment. While phosphatidylinositol 3-kinase (PI3K) inhibition as a Treg-targeted treatment has been implicated in animal models, its effects on human Tregs and on the potential impairment of effector T cells are required to be clarified for successful cancer immunotherapy.

**Methods:**

The impact of a selective-PI3K inhibitor ZSTK474 with or without anti-programmed cell death 1 (PD-1) monoclonal antibody on Tregs and CD8^+^ T cells were examined with in vivo animal models and in vitro experiments with antigen specific and non-specific fashions using peripheral blood from healthy individuals and cancer patients. Phenotypes and functions of Tregs and effector T cells were examined with comprehensive gene and protein expression assays.

**Results:**

Improved antitumor effects by the PI3K inhibitor in combination with ICB, particularly PD-1 blockade, were observed in mice and humans. Although administration of the PI3K inhibitor at higher doses impaired activation of CD8^+^ T cells as well as Tregs, the optimization (doses and timing) of this combination treatment selectively decreased intratumoral Tregs, resulting in increased tumor antigen-specific CD8^+^ T cells in the treated mice. Moreover, on the administration of the PI3K inhibitor with the optimal dose for selectively deleting Tregs, PI3K signaling was inhibited not only in Tregs but also in activated CD8^+^ T cells, leading to the enhanced generation of tumor antigen-specific memory CD8^+^ T cells which contributed to durable antitumor immunity. These opposing outcomes between Tregs and CD8^+^ T cells were attributed to the high degree of dependence on T cell signaling in the former but not in the latter.

**Conclusions:**

PI3K inhibitor in the combination with ICB with the optimized protocol fine-tuned T cell activation signaling for antitumor immunity via decreasing Tregs and optimizing memory CD8^+^ T cell responses, illustrating a promising combination therapy.

## Background

Cancer immunotherapy has emerged as a new class of cancer treatment with enormous clinical efficacy, even at advanced stages of disease. Immune checkpoint blockade (ICB), for example, by targeting cytotoxic T-lymphocyte associated antigen 4 (CTLA-4) and/or programmed cell death 1/programmed cell death ligand 1 (PD-1/PD-L1) with monoclonal antibodies (mAbs), can induce/resurge T cell responses against tumor cells, resulting in durable clinical benefit in multiple cancer types.^1 2^ According to the cancer immunoediting hypothesis, cancers reduce their expression of immunogenic molecules and employ multiple immune suppressive mechanisms including the expression of immune checkpoint molecules to establish an immunosuppressive tumor microenvironment (TME) and thus escape immunosurveillance.^3 4^ Hence, ICB can yield valuable clinical responses, yet more than half of the treated patients fail to respond to this therapy.^5^ This limited success is explained by the involvement of multiple immunosuppressive mechanisms, stressing the importance of developing combinatorial strategies targeting the various different suppressive mechanisms in the TME.^6^

In addition to immune checkpoint molecules, immune suppressive cells such as regulatory T cells (Tregs) play crucial roles in the immune suppressive TME.^7–9^ CD4^+^ Tregs expressing the transcription factor forkhead box P3 (FOXP3) are indispensable for the maintenance of immunological self-tolerance and homeostasis.^8^ However, FOXP3^+^CD4^+^ Tregs are also abundant in tumor tissues,^7–9^ and their depletion augments spontaneous and vaccine-induced T cell antitumor immune responses in animal models.^7^ In humans, increased numbers of FOXP3^+^CD4^+^ Tregs and, in particular, decreased ratios of CD8^+^ T cells to FOXP3^+^CD4^+^ Tregs among tumor-infiltrating lymphocytes (TILs), are significantly correlated with poor prognosis in various types of cancer.^7 9^ Therefore, Tregs are an attractive therapeutic target for cancer immunotherapy.^7^

Phosphatidylinositol 3-kinases (PI3Ks), a family of lipid kinases that phosphorylates phosphatidylinositol, are classified into three classes based on their primary structure and substrate specificity.^10^ The class I PI3Ks consist of PI3Kα, PI3Kβ, PI3Kδ (class IA) and PI3Kγ (class IB).^10^ PI3Kα and PI3Kβ are expressed ubiquitously whereas PI3Kδ and PI3Kγ are expressed mainly in leukocytes.^10^ Class I PI3K signaling is often activated in tumor cells and plays important roles in their proliferation and survival.^11^ Inhibitors against class I PI3K to directly attack tumor cells therefore are thought to be a promising cancer therapy.^11^ We have previously reported a selective PI3K inhibitor, ZSTK474, which inhibits all of the four class I PI3K isoforms, with a 3.5-fold to 10-fold higher specificity for PI3Kδ over the other PI3K isoforms, but not other 139 protein kinases tested.^12 13^ In T cells, PI3Kδ is highly expressed and transduces signals from the T-cell receptor (TCR), costimulatory receptors and cytokine receptors, thereby contributing to T cell differentiation, survival, and activation.^14^ Inactivation of PI3Kδ leads a decreased frequency and less suppressive capacity of Tregs, resulting in the activation of CD8^+^ T cell responses and subsequent tumor regression.^15–17^ Therefore, PI3Kδ inhibitors may be good candidates for combination therapy with ICB therapies such as PD-1 blockade. However, as PI3K signaling is also essential in effector T cell function such as memory T cell generation,^18–20^ complete PI3Kδ inactivation impairs CD8^+^ T cell function, canceling any advantages via Treg impairment.^21^ Therefore, the impact of PI3Kδ inhibitors on effector T cells in combination with ICB needs to be clarified for developing the combination.

Here, we show the combination of the PI3K inhibitor ZSTK474 and anti-PD-1 mAb as a novel cancer immunotherapy to achieve decreased Treg function and augmented effector T cell function with an optimized protocol. The optimized combination treatment provided a far stronger and durable inhibition of tumor growth. Additionally, the combination treatment increased tumor-specific CD8^+^ memory T cells via modulating PI3K signaling. Our data shed new light on the importance of optimizing the treatment protocol in combination cancer immunotherapy in which reagents that target T cell activation signals are employed.

## Materials and methods

### Cell lines and mice

CMS5a is a 3-methylcholanthrene-induced fibrosarcoma cell line of BALB/c origin. CMS5a-NY-ESO-1 is a cell line derived from CMS5a stably transfected with New York esophageal squamous cell carcinoma 1 (NY-ESO-1).^22^ B16F0 is a naturally developed malignant melanoma cell line in C57BL/6 mice. CMS5a, CMS5a-NY-ESO-1 and B16F0 were maintained in RPMI-1640 supplemented with 10% fetal bovine serum and 4 mmol/L of L-glutamine (CSTI, Miyagi, Japan) and were used after confirming that they were negative for *Mycoplasma*, as determined by testing with a PCR Mycoplasma Detection Kit (TaKaRa) according to the manufacturer’s instructions. Female BALB/c and C57BL/6 mice aged 6–7 weeks old were purchased from CLEA Japan (Tokyo, Japan). All mice were maintained in a specific pathogen-free facility at National Cancer Center Japan (Tokyo/Chiba, Japan).

### Antibodies and reagents

Antibodies used in this study are listed in online supplemental table S1. Peptides to stimulate T cells and major histocompatibility complex (MHC)-peptide tetramers to detect antigen-specific CD8^+^ T cells are listed in online supplemental table S2. ZSTK474 was synthesized by the R&D center of Zenyaku Kogyo (Tokyo, Japan) and Idelalisib was purchased from Selleck Chemicals (Houston, Texas, USA). These compounds were dissolved in dimethyl sulfoxide (DMSO) for in vitro experiments. ZSTK474 selectively inhibits PI3Kδ: it shows no or limited inhibition against more than 200 protein kinases (online supplemental table S3).^12 13^ Although ZSTK474 also inhibited mammalian target of rapamycin (mTOR) in kinase inhibition assays, its inhibition activity was 80 times higher against PI3Kδ than mTOR, indicating that ZSTK474 is a selective PI3K inhibitor with high specificity against PI3Kδ.^13^

10.1136/jitc-2020-002279.supp1 Supplementary data

### In vivo animal model

Details of animal experiments are described in online supplemental materials and methods.

10.1136/jitc-2020-002279.supp2 Supplementary data

### Murine cell isolation and staining

Details of the protocol for isolation and staining of murine cells to analyze T cell populations by flow cytometry are described in online supplemental materials and methods.

### In vitro CD3/CD28 stimulation

Details of the in vitro CD3/CD28 stimulation for T cell proliferation assay are described in online supplemental materials and methods.

### In vitro sensitization of antigen-specific CD8^+^ T cells

Details of the protocol for in vitro sensitization of antigen-specific CD8^+^ T cells by peptide antigen are described in online supplemental materials and methods.

### Human cell staining

Details of the protocol for staining human peripheral blood mononuclear cells (PBMCs) to analyze T cell populations by flow cytometry are described in online supplemental materials and methods.

### Phospho-flow cytometry

Details of the flow cytometric analysis of phspho-proteins in T cells are described in online supplemental materials and methods.

### Immunoblotting

Details of the immunoblot analysis are described in online supplemental materials and methods.

### RNA-seq analysis

Details of the RNA-seq analysis are described in online supplemental materials and methods.

### Quantitative real-time PCR

Details of the real-time quantitative RT-PCR analysis are described in online supplemental materials and methods.

### Statistics

Significance of differences between groups was determined with Excel and GraphPad Prism V.5 software using one-way analysis of variance (ANOVA) with post hoc Tukey’s test or Dunnett’s test, two-way ANOVA with post hoc Dunnett’s test, Kruskal-Wallis post hoc Steel’s test, unpaired two-tailed Student's t-test, Gehan-Breslow-Wilcoxon test and log rank test. Values of p<0.05 were considered statistically significant.

## Results

### Treatment with the PI3K inhibitor ZSTK474 activates antitumor immunity

**Figure 1:**
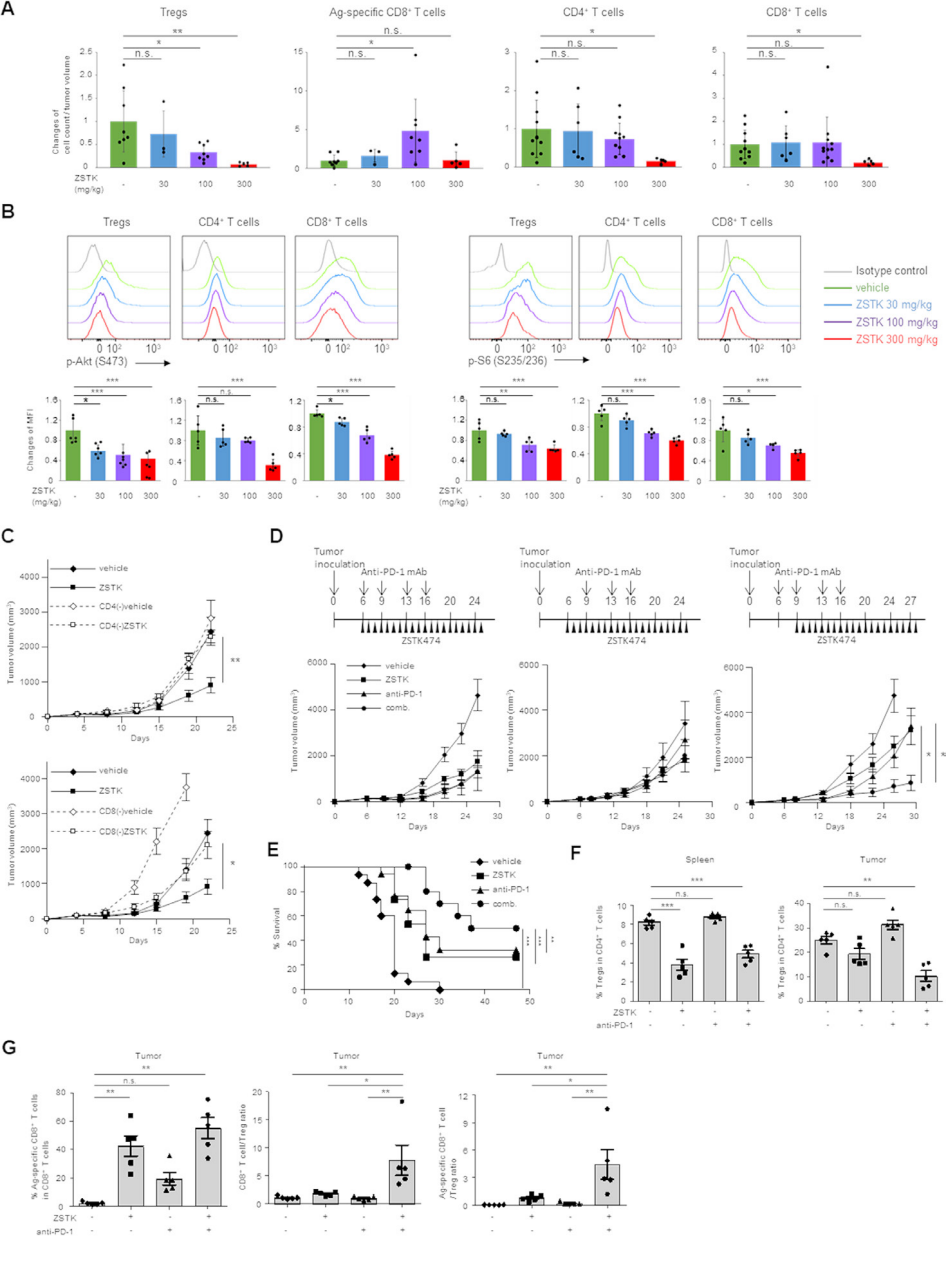
Treatment of ZSTK474 alone or in combination with anti-PD-1 mAb activates antitumor immunity via Treg suppression. (A) The relative changes of intratumoral Treg, tumor antigen-specific CD8^+^ T cell, CD4^+^ T cell and CD8^+^ T cell counts per tumor volume (mm^3^) by ZSTK474 treatment in mice-bearing CMS5a-NY-ESO-1. ZSTK474 was administrated at 30, 100 or 300 mg/kg once a day from day 6 to 13. T cells were collected from tumors 14 days after tumor inoculation and were subjected to flow cytometry. Data are means±SD. (B) Phosphorylation status of Akt at S473 (p-Akt) and S6 at S235/236 (p–S6) in Tregs, helper CD4^+^ T cells and CD8^+^ T cells in DLNs of mice-bearing CMS5a-NY-ESO-1 treated with ZSTK474 at 30, 100 or 300 mg/kg once a day from day 6 to 12. DLNs were collected from mice 2 hours after the last administration and T cells in DLNs were subjected to flow cytometry. Representative flow cytometry histograms (upper panels) and summaries of mean fluorescence (MFI) of p-Akt and p-S6 in Tregs, helper CD4^+^ T cells and CD8^+^ T cells are shown. (C) Tumor growth inhibition of CMS5a-NY-ESO-1 tumors by ZSTK474 treatment (n=6 per group) in CD4^+^ or CD8^+^ T cell-deleted mice. (D) Antitumor effects of the combination therapy with ZSTK474 and anti-PD-1 mAb (three different protocols) in CMS5a-NY-ESO-1 model (n=8 per group). ZSTK474 and anti-PD-1 mAb were administered as shown in each upper panel. (E) Survival curves of CMS5a-NY-ESO-1-bearing mice treated with or without ZSTK474 (once a day from day 9 to 28) and/or anti-PD-1 mAb (day 6, 9, 13 and 16). (F, G) Mice-bearing CMS5a-NY-ESO-1 were treated with or without ZSTK474 (once a day from day 9 to 15) and/or anti-PD-1 mAb (day 6, 9 and 13). T cells were collected from spleens and tumors at 16 days after tumor inoculation and were subjected to flow cytometry. The frequencies of Tregs in CD4^+^ T cells in spleens (left) and tumors (right in F), the frequencies of NY-ESO-1 specific CD8^+^ T cells (left), CD8^+^ T cell:Treg ratio (middle) and NY-ESO-1-specific CD8^+^ T cell:Treg ratio (right in G) in tumors. Data are means±SE. Statistical analyses were performed by Dunnett’s test (A, B, D, F, G), Student’s t-test (C) and Gehan-Breslow-Wilcoxon test (E). These experiments were performed independently at least two to three times with similar results. *P<0.05; **p<0.01; ***p<0.001. DLNs, draining lymph nodes; mAbs, monoclonal antibodies; ns, not significant; Treg, regulatory T cells.

Since complete PI3K inactivation in T cells impairs not only Tregs but also effector T cells, canceling the benefits of Treg disruption,^21^ we first investigated the optimal protocol for ZSTK474 to augment antitumor immune responses via decreasing Tregs and activating effector T cells such as CD8^+^ T cells. To explore the potential of the combination treatment with ZSTK474 and PD-1 blockade, we employed tumor cell lines (CMS5a and B16F0) that are resistant to PD-1 blockade.^23^ Mice-bearing CMS5a cells stably expressing a tumor antigen, NY-ESO-1 (CMS5a-NY-ESO-1) were treated with ZSTK474. ZSTK474 treatment exhibited antitumor effects and decreased intratumoral Tregs in a dose-dependent manner. However, high-dose (300 mg/kg) treatment of ZSTK474, but not low (30 mg/kg) or intermediate-dose (100 mg/kg) treatment, significantly decreased all CD4^+^ and CD8^+^ T cell subsets. While tumor antigen (NY-ESO-1)-specific CD8^+^ T cells were increased at the intermediate dose, the treatment of high-dose ZSTK474 failed to increase NY-ESO-1-specific CD8^+^ T cells (figure 1A and online supplemental figure S1A, B).

10.1136/jitc-2020-002279.supp3 Supplementary data

We next examined the effects of ZSTK474 treatment on PI3K signaling in T cells. Treatment with ZSTK474 to mice-bearing CMS5a-NY-ESO-1 reduced phosphorylation of PI3K signaling molecules including Akt and S6, but not a PI3K upstream molecule SH2 domain–containing leukocyte protein of 76 kDa (SLP-76), in Tregs, CD4^+^ T cells and CD8^+^ T cells in a dose dependent manner (figure 1B and online supplemental figure S1C). Therefore, the treatment of high dose ZSTK474 such as 300 mg/kg significantly decreases the entire CD4^+^ and CD8^+^ T cell subsets including Tregs through inhibiting PI3K signaling in T cells whereas the treatment of intermediate dose ZSTK474 such as 100 mg/kg selectively decreases Tregs through inhibiting PI3K signaling at moderate degree, that leaves weak-to-intermediate signaling of PI3K to allow helper CD4^+^ and CD8^+^ T cell proliferation.

Since intermittent regimen with PI3K inhibitor is comparably or more effective than continuous regimen,^24^ we addressed whether the intermittent dosing with ZSTK474 showed superior antitumor effects and T cell activation compared with the continuous dosing with ZSTK474. Mice-bearing CMS5a-NY-ESO-1 were treated with ZSTK474 at the intermittent or the continuous dosing schedule. Both dosing schedules exhibited similar antitumor effects, Treg suppression, induction of NY-ESO-1-specific CD8^+^ T cells and body weight loss. Then, we employed the continuous dosing schedule for further experiments (online supplemental figure S1D–F). At the continuous dosing, Tregs were suppressed from day 10, and then NY-ESO-1-specific CD8^+^ T cells were induced at day 14 (online supplemental figure S1G), indicating that ZSTK474 treatment impairs Treg suppression, consequently inducing the activation of tumor antigen-specific CD8^+^ T cells.

We confirmed whether ZSTK474 treatment at the intermediate dose (100 mg/kg) exhibited tumor growth inhibition via T cell activation. Mice-bearing CMS5a-NY-ESO-1 were treated with ZSTK474 and depleted CD4^+^ T cells or CD8^+^ T cells using anti-CD4 mAb or anti-CD8 mAb, respectively. ZSTK474 treatment significantly inhibited tumor growth, but depletion of either CD4^+^ T cells or CD8^+^ T cells abrogated the antitumor effects of ZSTK474, indicating that both CD4^+^ T cells and CD8^+^ T cells contribute to tumor growth inhibition by ZSTK474 (figure 1C).

### Treatment with a combination of ZSTK474 and anti-PD-1 mAb mediates strong antitumor effects associated with decreased Tregs and increased tumor antigen-specific CD8^+^ T cells

As ZSTK474 alone did not achieve the complete eradication of tumors and ZSTK474 treatment significantly induced PD-1 expression on intra-tumoral CD8^+^ T cells (online supplemental figure S1H), we investigated antitumor effects of a combination treatment with ZSTK474 and anti-PD-1 mAb. Mice-bearing CMS5a-NY-ESO-1 were treated with ZSTK474 (100 mg/kg) and anti-PD-1 mAb either alone or in combination. Anti-CTLA-4 mAb (ipilimumab) leads to depletion of CTLA-4-expressing Tregs and yields higher response rates in combination with anti-PD-1 mAb (nivolumab) in some patients with malignant melanoma, kidney cancer and lung cancer.^25^ In this combination therapy, nivolumab followed by ipilimumab appears to be more clinically beneficial compared with the opposite order.^26^ Thus, we explored antitumor effects of three different protocols of the combination treatment: 1) ZSTK474 and anti-PD-1 mAb were concurrently administered, (2) ZSTK474 single treatment was started and then anti-PD-1 mAb plus ZSTK474 combination treatment was followed, and (3) anti-PD-1 mAb alone was started and then ZSTK474 plus anti-PD-1 mAb combination treatment was followed (figure 1D). Only one protocol (#3), in which anti-PD-1 mAb alone was started and then ZSTK474 plus anti-PD-1 mAb combination treatment was followed, showed a significantly efficient tumor growth inhibition and improved survival compared with either ZSTK474 or anti-PD-1 mAb alone (figure 1D and E). In the optimal protocol, Tregs in tumors and spleens were significantly reduced by ZSTK474 treatment alone or in combination with anti-PD-1 mAb, although the difference with Tregs in tumors between vehicle and ZSTK474 alone groups were not statistically significant (figure 1F and online supplemental figure S2A, B). In the other protocols (#1 and #2), Tregs in tumors were also significantly reduced by ZSTK474 treatment alone, but adding anti-PD-1 mAb to ZSTK474 treatment impaired the reduction of Tregs by ZSTK474 (online supplemental figure S3A). Tumor antigen (NY-ESO-1)-specific CD8^+^ T cells were significantly increased in tumors by ZSTK474 treatment alone and further augmented in combination with anti-PD-1 mAb in any protocols (figure 1G and online supplemental figure S2C,S3B). Accordingly, in the optimal protocol, but not the other two protocols, intratumoral CD8^+^ T cell:Treg ratios and tumor antigen (NY-ESO-1)-specific CD8^+^ T cell:Treg ratios were significantly increased in the combination treatment group compared with either treatment alone (figure 1G and online supplemental figure S3C), indicating that the immune balance (effector vs inhibitory) in the TME is shifted toward effector T cell responses against the tumor by the optimal combination treatment.

10.1136/jitc-2020-002279.supp4 Supplementary data

10.1136/jitc-2020-002279.supp5 Supplementary data

Since ZSTK474 inhibits all of the four class I PI3K isoforms, with a 3.5-fold to 10-fold higher specificity for PI3Kδ over the other PI3K isoforms, α, β and γ,^13^ we compared Treg suppression activity of ZSTK474 with that of PI3Kδ specific inhibitor, Idelalisib in the optimal protocol. Idelalisib treatment comparably decreased Tregs compared with ZSTK474. However, tumor antigen (NY-ESO-1)-specific CD8^+^ T cells was not efficiently activated by the treatment with Idelalisib, suggesting that a pan-PI3K inhibitor ZSTK474 may be superior to a PI3Kδ specific inhibitor Idelalisib in activating effector T cells (online supplemental figure S3D).

The strong antitumor efficacy in the optimal protocol prompted us to examine the potential of the combination treatment in less immunogenic tumors such as B16F0 that are resistant to PD-1 blockade.^23^ Mice-bearing B16F0 cells were treated with ZSTK474 and anti-PD-1 mAb either alone or in combination. The combination treatment exhibited a significantly superior tumor growth inhibition and activation of T cell responses against B16F0 melanoma, a PD-1 blockade-resistant tumor (online supplemental figure S4A, B). We also examined the antitumor effects by the combination treatment of ZSTK474 at high dose (300 mg/kg) with anti-PD-1 mAb on tumor growth. As expected, the combination treatment failed to augment the antitumor effects by ZSTK474 alone (online supplemental figure S4C). Taken together, the PI3K inhibitor ZSTK474 at the intermediate dose (100 mg/kg) decreases Tregs and increases antitumor CD8^+^ T cells, and the antitumor CD8^+^ T cell responses are further augmented by pretreatment with anti-PD-1 mAb, resulting in a far stronger tumor growth inhibition by the combination treatment.

10.1136/jitc-2020-002279.supp6 Supplementary data

### ZSTK474 augments antigen-specific CD8^+^ T cell induction/activation via inhibiting Tregs in humans

**Figure 2:**
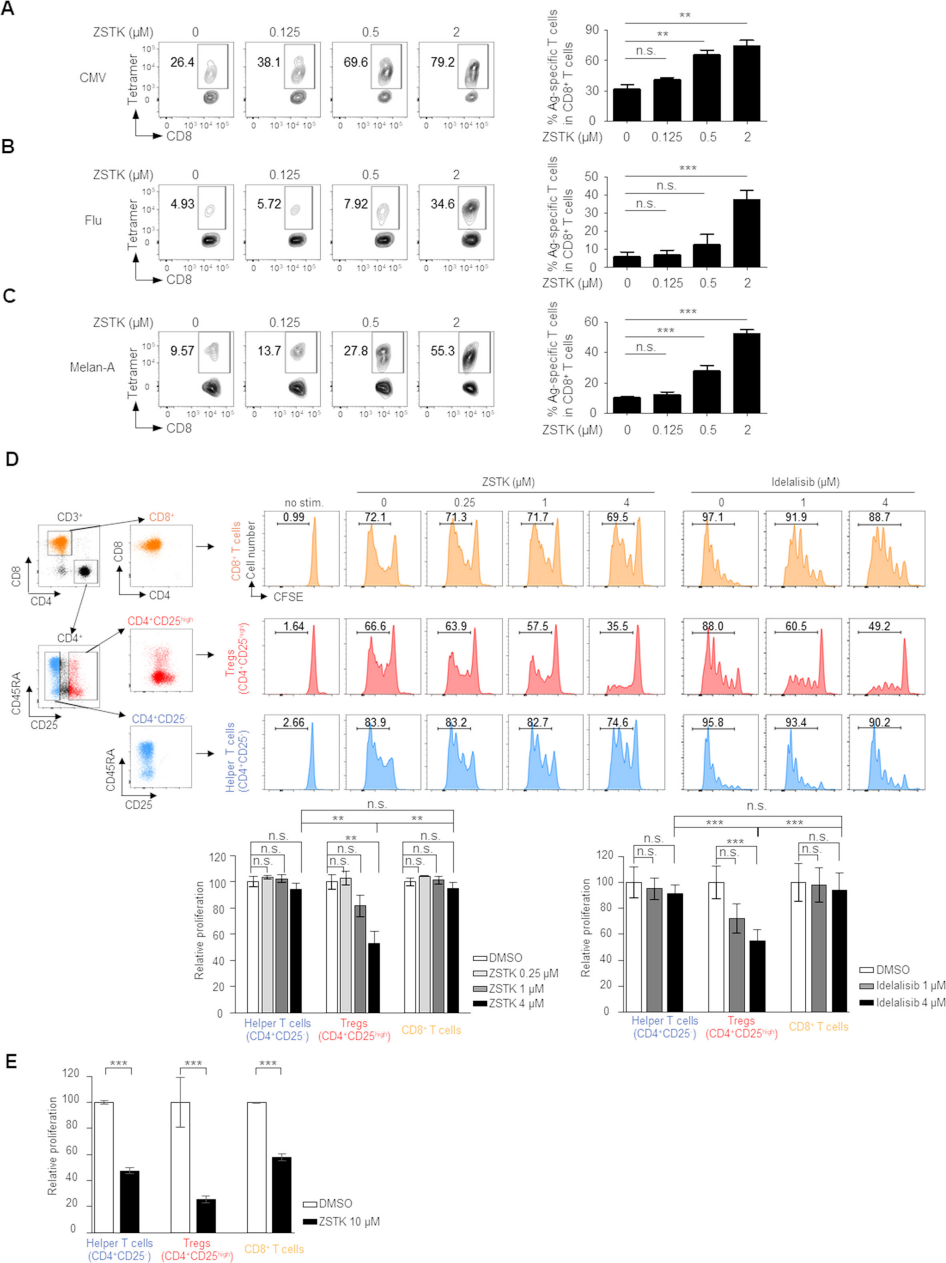
Inhibition of PI3K signaling by ZSTK474 increases induction of antigen-specific CD8^+^ T cells by selectively inhibiting Tregs in humans. (A–C) CD8^+^ T cell responses to CMV (A), Influenza (B) or Melan-A (C) peptides treated with the indicated doses of ZSTK474. Antigen-specific CD8^+^ T cells in peripheral blood mononuclear cells (PBMCs) from healthy individuals were detected by MHC/peptide multimers. Representative flow cytometric analysis (left) and summaries of triplicate data (right). The numbers in the panels indicate the frequencies of gated CD8^+^ T cells. These experiments were performed independently at least two to three times with similar results. (D, E) Tregs (CD4^+^CD25^high^), helper CD4^+^ T cells (CD4^+^CD25^-^) and CD8^+^ T cells were prepared from human PBMCs and labeled with CFSE. Proliferation was examined by CFSE dilution after stimulation with anti-CD3/anti-CD28 mAb for 4 days with or without the indicated dose of ZSTK474 or Idelalisib. A representative staining (upper panels in D) and summaries of three independent experiments (lower panles in D and E). The numbers in the panels indicate the frequencies of proliferated T cells. Data are means±SE. Statistical analyses were performed by Dunnett’s test (A–C) and Tukey’s test (D, E). *P<0.05; **p<0.01; ***p<0.001. CMV, cytomegalovirus; mAb, monoclonal antibody; ns, not significant; Treg, regulatory T cell.

Since ZSTK474 augmented antitumor CD8^+^ T cell responses in murine models, we next asked whether it would also increase antigen-specific T cell responses in humans. There are at least two types of tumor antigens: (1) tumor-specific antigens (TSAs), which are either oncogenic viral proteins or abnormal proteins stemming from somatic mutations (neoantigens), and (2) tumor-associated antigens (TAAs), which are highly or aberrantly expressed normal proteins.^27^ As surrogate TSAs, we employed cytomegalovirus (CMV) and influenza virus (Influenza), and assessed antigen-specific CD8^+^ T cell responses against CMV and Influenza using CMV/HLA-A*0201 and Influenza/HLA-A*0201 tetramers. CMV- and Flu-specific CD8^+^ T cells were elicited by the stimulation with each cognate antigen, and this was significantly increased by ZSTK474 treatment (figure 2A and B). We further analyzed whether ZSTK474 amplified TAA-specific CD8^+^ T cell responses using a representative TAA, Melan-A (also known as MART-1). Melan-A-specific CD8^+^ T cells were significantly increased by treatment with ZSTK474 (figure 2C). Together, ZSTK474 augments TSA and TAA-specific CD8^+^ T cell responses in humans.

To examine whether ZSTK474 increased antigen-specific CD8^+^ T cell responses directly or indirectly via inhibiting Treg suppression, we sorted Tregs (CD4^+^CD25^high^), helper CD4^+^ T cells (CD4^+^CD25^-^) and CD8^+^ T cells from human PBMCs and evaluated the effect of ZSTK474 on proliferation of each T cell population. As a control, PI3Kδ inhibitor, Idelalisib of which kinase inhibition activity for PI3Kδ is comparable to ZSTK474, was used. While helper CD4^+^ T cells and CD8^+^ T cells vigorously proliferated in response to anti-CD3/anti-CD28 mAb stimulation, ZSTK474 strongly inhibited the proliferation of Tregs compared with helper CD4^+^ or CD8^+^ T cells as well as Idelalisib (figure 2D). As the treatment of high dose ZSTK474 in vivo significantly reduced CD4^+^ T cells and CD8^+^ T cells in addition to Tregs, we examined whether high concentration (10 µM) of ZSTK474 inhibited proliferation of not only Tregs but also CD4^+^ T cells and CD8^+^ T cells in vitro. High concentration of ZSTK474 significantly inhibited proliferation of CD4^+^ T cells and CD8^+^ T cells as well as Tregs (figure 2E). Thus, the optimized dosage of ZSTK474 selectively inhibits Treg proliferation due to PI3Kδ inhibition, but not other T cells such as helper CD4^+^ T cells and CD8^+^ T cells.

### ZSTK474 inhibits PI3K signaling in T cell subsets

**Figure 3:**
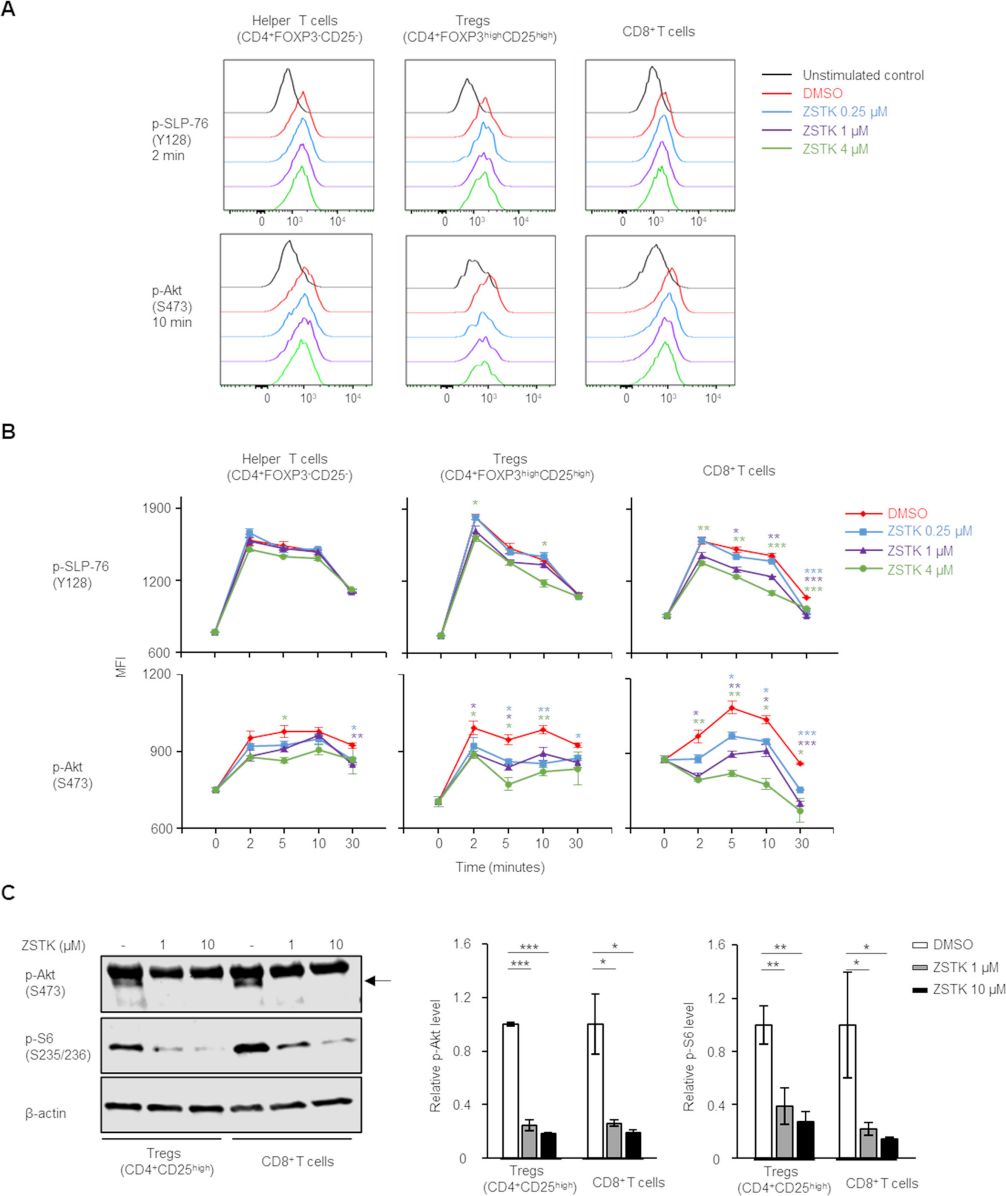
ZSTK474 inhibits PI3K signaling not only in Tregs but also in CD8^+^ T cells. (A, B) Phosphorylation status of SLP-76 at Y128 (p-SLP-76) and Akt at S473 (p-Akt) after stimulation with anti-CD3/anti-CD28 mAb at the indicated time points with the indicated doses of ZSTK474. Representative flow cytometry histograms (A) and summaries of mean fluorescence (MFI) of p-SLP-76 and p-Akt (B) in Tregs, helper CD4^+^ T cells and CD8^+^ T cells. Data are means±SE. (C) Phosphorylation of Akt at S473 (p-Akt) and S6 at S235/236 (p–S6) after stimulation with anti-CD3/anti-CD28 mAb for 20 min with or without ZSTK474 (1 or 10 µM). Representative pictures of immunoblot analyses (left) and the average of quantification values of bands (right). Data are means±SD. Statistical analyses were performed by Student’s t-test (B), and Dunnett’s test (C). *P<0.05; **p<0.01; ***p<0.001. mAb, monoclonal antibody.

The different effects of ZSTK474 on Tregs, helper CD4^+^ T cells and CD8^+^ T cells led us to examine PI3K signaling in each T cell type in response to ZSTK474 in vitro. Human PBMCs were stimulated with anti-CD3/anti-CD28 mAb with or without ZSTK474, and phosphorylation levels of the TCR signaling molecules in the stimulated Tregs, helper CD4^+^ T cells and CD8^+^ T cells were examined with flow cytometry. While the stimulation with anti-CD3/anti-CD28 mAb induced the phosphorylation of the TCR signaling molecules including SLP-76 (p-SLP-76), Akt (p-Akt) and S6 (p-S6) in helper CD4^+^ T cells and CD8^+^ T cells as well as Tregs, ZSTK474 significantly reduced the phosphorylation of PI3K signaling molecules including Akt in Tregs, helper CD4^+^ T cells and CD8^+^ T cells in a dose dependent manner (figure 3A, B). The upstream molecule of PI3K, SLP-76 was slightly reduced in Tregs and CD8^+^ T cells, but not in helper CD4^+^ T cells by ZSTK474 (figure 3A, B). We further confirmed the activation levels of PI3K signaling on ZSTK474 treatment with immunoblotting in Tregs and CD8^+^ T cells in which the remarkable inhibition of PI3K signaling was observed with flowcytometry. ZSTK474 completely inhibited the phosphorylation of Akt in Tregs and CD8^+^ T cells at any concentrations tested (1 µM and 10 µM) although the phosphorylation of S6 was inhibited in Tregs and CD8^+^ T cells in a dose-dependent manner (figure 3C and online supplemental figure S5). In the inhibition of cell proliferation, ZSTK474 selectively inhibited proliferation of Tregs at 1 µM, whereas high concentration (10 µM) of ZSTK474 significantly inhibited helper CD4^+^ T cells and CD8^+^ T cells as well as Tregs. These results suggest that PI3K signaling is essential for Tregs while inactivation, though leaving weak-to-intermediate signaling, of PI3K signaling by ZSTK474 treatment at intermediate concentration such as 1 µM may allow the proliferation of helper CD4^+^ T cells and CD8^+^ T cells.

10.1136/jitc-2020-002279.supp7 Supplementary data

### Inhibition of PI3K signaling by ZSTK474 in CD8^+^ T cells enhances memory T cell differentiation

**Figure 4:**
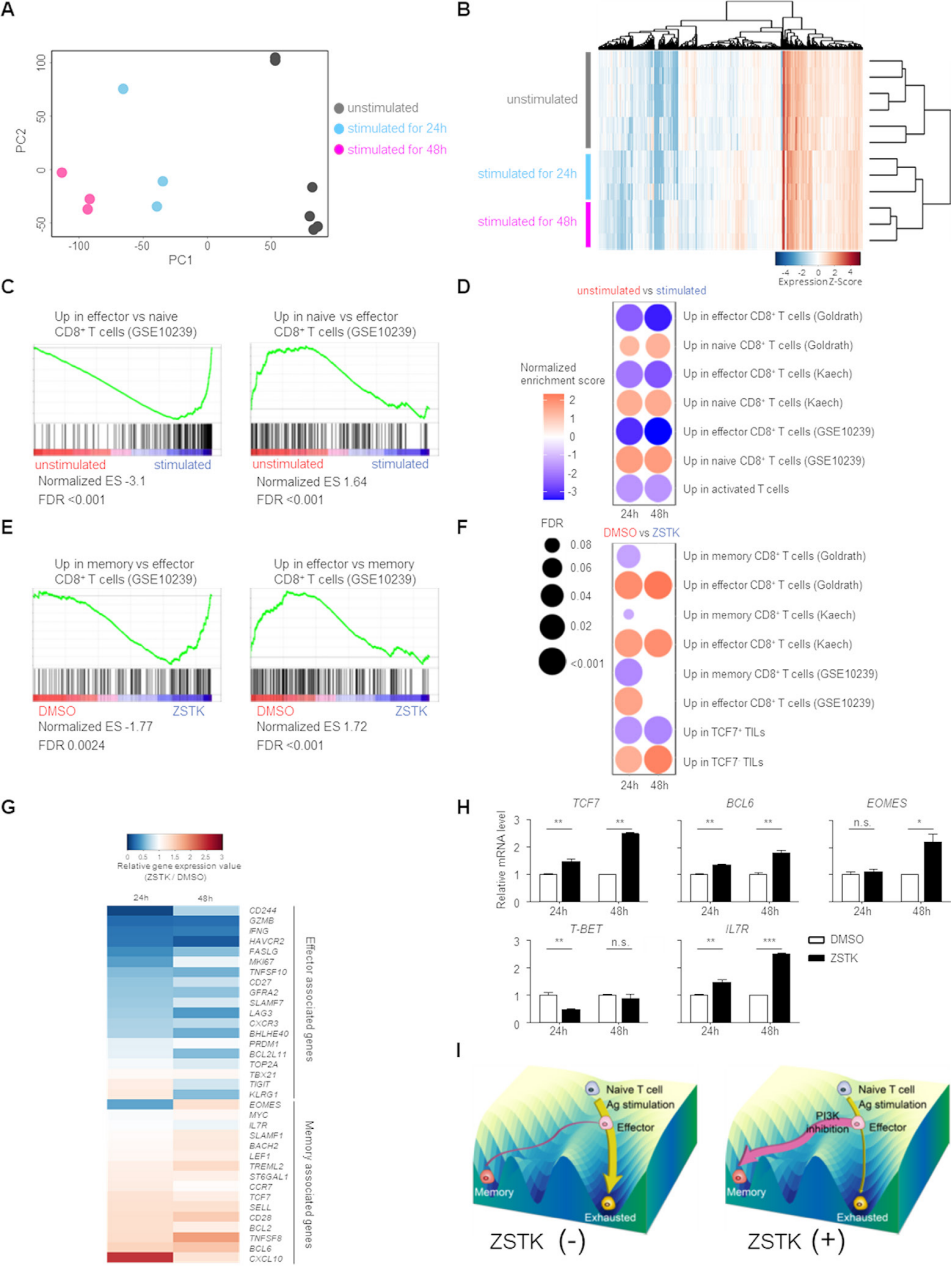
CD8^+^ T cells treated with ZSTK474 exhibits memory T cell-like gene expression profiles. (A–G) Naive CD8^+^ T cells (CD45RA^+^CCR7^+^) prepared from PBMCs of three healthy individuals were stimulated with anti-CD3/anti-CD28 mAb for 24 and 48 hours with or without ZSTK474 (1 µM) and subjected to RNA-seq analysis. (A, B) Principal component analysis (A) and unsupervised hierarchical clustering (B) of gene expression profiles in unstimulated naive CD8^+^ T cells and CD8^+^ T cells stimulated for 24 or 48 hours. (C, D) Representative gene-set enrichment analysis (GSEA) plots (C) and summaries of GSEA (D). The enrichment of naive or effector CD8^+^ T cell signatures in unstimulated CD8^+^ T cells versus stimulated CD8^+^ T cells is shown. Color scale represents the enrichment score in unstimulated CD8^+^ T cells compared with that in stimulated CD8^+^ T cells. Circle size indicates the false discovery rate (FDR) in (D). (E, F) Representative GSEA plots (E) and summaries of GSEA (F). The enrichment of effector or memory CD8^+^ T cell signatures in stimulated CD8^+^ T cells without vs with ZSTK474 is shown. (G) Heatmap showing relative gene expression of effector and memory T cell-associated genes in CD8^+^ T cells stimulated with vs without ZSTK474 for 24 or 48 hours. (H) Real-time quantitative RT-PCR analysis of memory T cell-associated transcription factors. mRNA levels of *TCF-7*, *BCL-6*, *EOMES*, *T-BET* and *IL7R* were measured. Data are the average of triplicate assays and are means±SE. These experiments were performed independently at least twice to three times with similar results. (I) Schematic illustration of Waddington’s landscape model showing differentiation of naive CD8^+^ T cells into effector and memory T cells by antigen stimulation in the presence or absence of ZSTK474, a PI3K inhibitor. Statistical analyses were performed by Student’s t-test (H). *P<0.05; **p<0.01; ***p<0.001. mAb, monoclonal antibody; ns, not significant; PBMCs, peripheral blood mononuclear cells; TILs, tumor-infiltrating lymphocytes.

Based on the above results, we investigated how inhibition of PI3K signaling by ZSTK474 influenced CD8^+^ T cells by comprehensively analyzing gene expression profiles. Naive CD8^+^ T cells (CD45RA^+^CCR7^+^) prepared from human PBMCs were stimulated with anti-CD3/anti-CD28 mAb in the presence or absence of ZSTK474 for 24 or 48 hours and were subjected to RNA-seq analysis. Principal component analysis and cluster analysis revealed that the gene expression profiles of naive CD8^+^ T cells and CD8^+^ T cells stimulated with anti-CD3/anti-CD28 mAb for 24 or 48 hours were differently clustered (figure 4A, B). As expected, CD8^+^ T cells stimulated with anti-CD3/anti-CD28 mAb highly expressed effector T cell signature genes, whereas expression of naive T cell signature genes was significantly reduced in those cells compared with naive CD8^+^ T cells (figure 4C, D). On treatment of the stimulated CD8^+^ T cells with ZSTK474, memory T cell signature rather than effector T cell signature was significantly enriched in the stimulated CD8^+^ T cells with the gene expression profiles (figure 4E, F). These CD8^+^ T cells treated with ZSTK474 further increased the expression of a subset of genes associated with TCF7-expressing TILs, which reportedly share molecular and functional similarities to memory cells.^28^ By contrast, a subset of genes highly expressed in TCF7-non-expressing TILs were detected in CD8^+^ T cells that were not treated with ZSTK474 (figure 4F). In accordance with these signature analyses, memory cell-related molecules were increased in CD8^+^ T cells treated with ZSTK474 compared with those not treated with ZSTK474, although effector cell-related genes were decreased in CD8^+^ T cells treated with ZSTK474 compared with those not treated with ZSTK474 (figure 4G). In particular, the expression of the key transcription factors in the differentiation of memory T cells including *TCF7*, *BCL6* and *EOMES* was significantly upregulated in ZSTK474-treated CD8^+^ T cells, while the expression of *T-BET*, which is an important transcription factor for promoting the terminal differentiation of effector T cells, was not enhanced in CD8^+^ T cells by ZSTK474 treatment (figure 4H).^20 28^ Therefore, ZSTK474 enhances the expression of memory cell-associated transcription factors early after T cell stimulation, leading to the predominant memory T cell differentiation rather than exhausted state during CD8^+^ T cell activation (illustrated in figure 4I).

**Figure 5:**
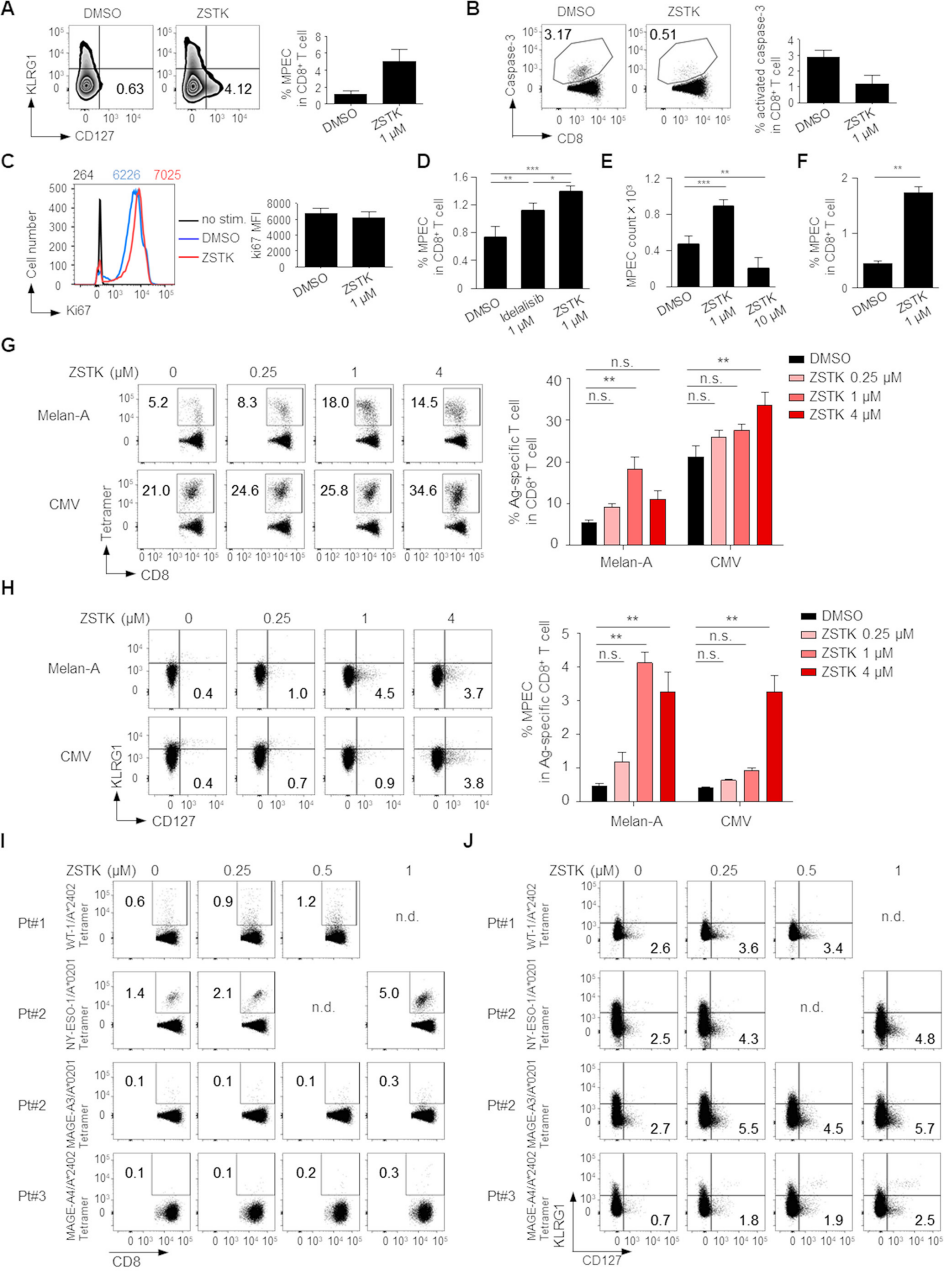
Inhibition of PI3K signaling by ZSTK474 in CD8^+^ T cells enhances memory T cell differentiation. (A–F) Naive CD8^+^ T cells (CD45RA^+^CCR7^+^) (A–E) and effector CD8^+^ T cells (CD45RA^-^CCR7^-^) (F) prepared from PBMCs of healthy individuals were stimulated with anti-CD3/anti-CD28 mAb for 8 days with or without the indicated doses of ZSTK474 or Idelalisib. Representative flow cytometry staining (left) and summary (right) of the frequencies of MPECs (KLRG1^-^CD127^+^) in CD8^+^ T cells (A), the frequencies of activated-caspase-3^+^CD8^+^ T cells in CD8^+^ T cells (B), the mean fluorescence intensity (MFI) of Ki67 (C), summary of the frequencies of MPECs (KLRG1^-^CD127^+^) in CD8^+^ T cells (D), absolute counts of MPEC per well in 96-well plates (E) and the frequencies of MPECs (KLRG1^-^CD127^+^) in CD8^+^ T cells (F). The numbers in the panels indicate the frequencies in CD8^+^ T cells (A, B) and the MFI of Ki67 in the indicated cells with the same color (C). (G, H) CD8^+^ T cells in PBMCs from healthy individuals were stimulated by X-irradiated APCs pulsed with Melan-A or CMV peptide with or without the indicated doses of ZSTK474 for 8 days. Representative flow cytometry staining (left) and summaries (right) of the frequencies of antigen-specific CD8^+^ T cells (G) and MPECs in antigen-specific CD8^+^ T cells (H). The numbers in the panels indicate the frequencies in CD8^+^ T cells (G) and the frequencies in antigen-specific CD8^+^ T cells (H). (I, J) CD8^+^ T cell responses against cancer antigens treated with the indicated doses of ZSTK474. CD8^+^ T cells in PBMCs from gastric cancer patients were cultured with or without ZSTK474 in the presence of X-irradiated CD4^−^CD8^−^ PBMCs as APCs pulsed with WT-1, NY-ESO-1, MAGE-A3 or MAGE-A4 peptide. Flow cytometry staining of antigen-specific CD8^+^ T cells (I) and MPECs in antigen-specific CD8^+^ T cells (J). The experiments (A–H) were performed independently at least two to three with similar results. Data are means±SE. Statistical analyses were performed by Dunnett’s test (D–H). *P<0.05; **p<0.01; ***p<0.001. APCs, antigen-presenting cells; CMV, cytomegalovirus; mAb, monoclonal antibody; MPECs, memory precursor effector cells; nd, no data; ns, not significant; PBMCs, peripheral blood mononuclear cells.

We further explored the influence of PI3K inhibition by ZSTK474 on CD8^+^ memory T cell differentiation. Naive CD8^+^ T cells (CD45RA^+^CCR7^+^) were prepared from human PBMCs and stimulated with anti-CD3/anti-CD28 mAb with or without ZSTK474 or Idelalisib for 8 days. ZSTK474 treatment significantly increased CD8^+^ memory precursor effector cells (MPECs)^29^ detected as KLRG1^-^CD127^+^ phenotype and decreased apoptosis, although the levels of proliferation marker Ki67 were similar regardless of ZSTK474 treatment (figure 5A–C). Idelalisib treatment significantly, although weaker than ZSTK474 treatment, increased MPECs (figure 5D). In helper CD4^+^ T cells, PI3Kα and PI3Kβ provide a redundant pathway to PI3Kδ.^16^ Although it remains fully elucidated that PI3Kα and PI3Kβ have roles in CD8^+^ T cells as well as helper CD4^+^ T cells, this could be a reason why pan-PI3K inhibitor ZSTK474 is more effective than PI3Kδ specific inhibitor Idelalisib to induce memory CD8^+^ T cells. We next examined MPEC induction by high concentration (10 µM) of ZSTK474 treatment. As expected, high concentration of ZSTK474 treatment significantly reduced the absolute counts of MPECs (figure 5E). Since memory T cells could differentiate from naive and effector T cells after antigen recognition,^30^ we examined whether ZSTK474 recalled MPECs from effector CD8^+^ T cells. Effector CD8^+^ T cells (CD45RA^-^CCR7^-^) were prepared from human PBMCs and stimulated with anti-CD3/anti-CD28 mAb with or without ZSTK474 for 8 days. ZSTK474 significantly increased MPEC generation from effector CD8^+^ T cells as well as naive CD8^+^ T cells (figure 5F). These results indicate that although we cannot completely rule out the possibility that the memory T cells induced by ZSTK474 could be differentiated from a few naive T cells contaminated in the sorted effector T cells, memory T cells are differentiated from naive and effector, probably early/juvenile effector, T cells. We also examined the influence of ZSTK474 on CD8^+^ memory T cell differentiation following antigen-specific T cell stimulation. To this end, CD8^+^ T cells from PBMCs were stimulated with X-irradiated (35 Gy) antigen-presenting cells (APCs, CD4^-^CD8^-^PBMCs) pulsed with Melan-A or CMV peptide overnight as previously described.^22^ Eight days later, Melan-A or CMV-specific CD8^+^ T cells stimulated by the cognate antigen were significantly increased by ZSTK474 treatment (figure 5G). More importantly, MPECs in the antigen-specific CD8^+^ T cell populations were also significantly enhanced by ZSTK474 treatment (figure 5H). To further examine the induction of TAA-specific memory T cells by ZSTK474 in cancer settings, we used PBMCs from three gastric cancer patients who exhibited humoral immune responses against WT-1, NY-ESO-1, MAGE-A3 or MAGE-A4 that were expressed by their own tumors. ZSTK474 treatment markedly increased both tumor antigen-specific CD8^+^ T cells and MPECs in these patients in vitro (figure 5I, J). Thus, ZSTK474 promotes the differentiation of activated CD8^+^ T cells into memory T cells via inhibition of PI3K signaling.

### The combination with ZSTK474 and anti-PD-1 mAb increases memory T cells and exhibits durable antitumor effects

**Figure 6:**
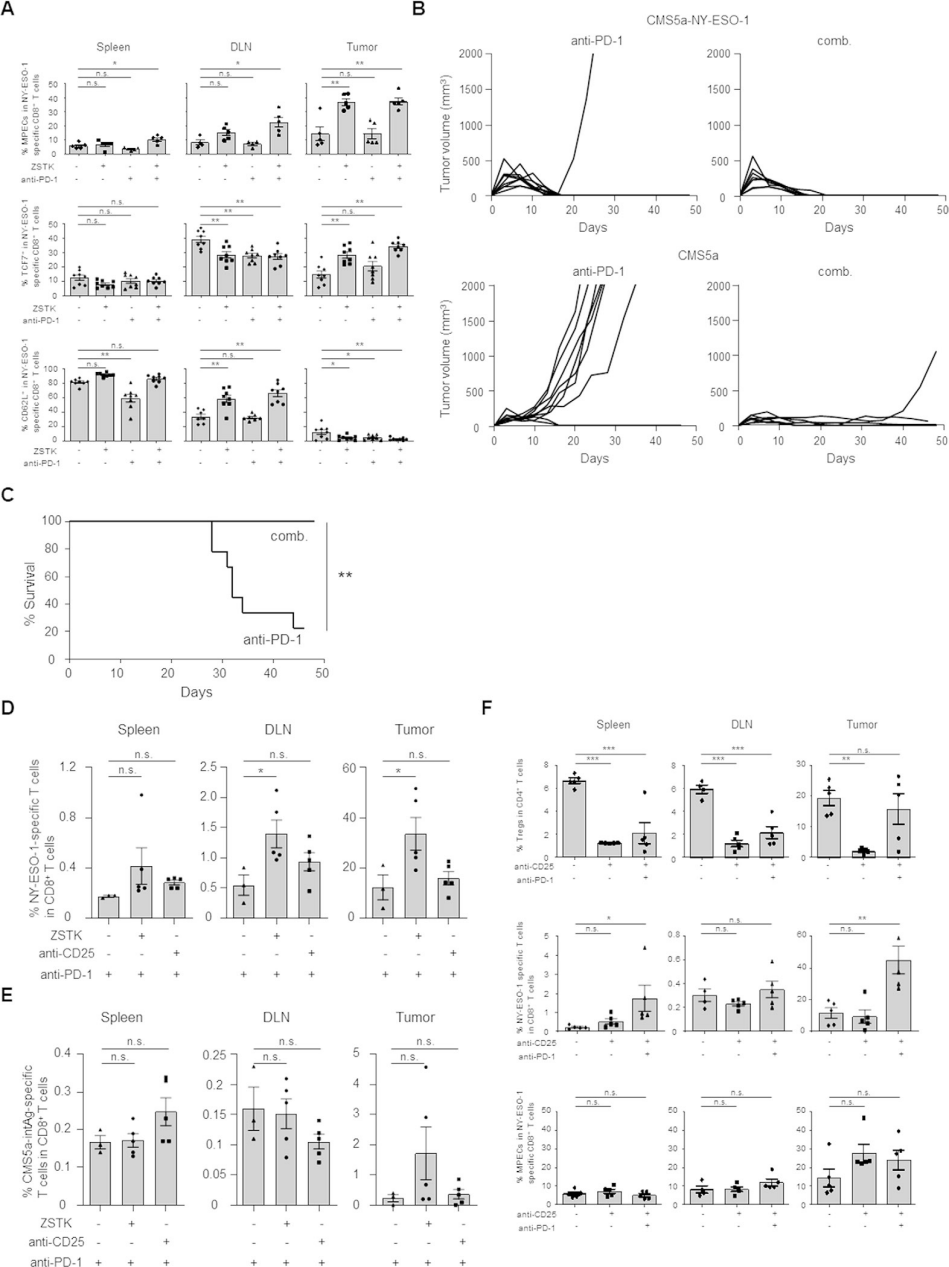
ZSTK474 but not anti-CD25 mAb in combination with anti-PD-1 mAb increases memory T cells in vivo, resulting in augmented durable antitumor effects. (A) Effects of ZSTK474 and anti-PD-1 mAb either alone or in combination on induction of MPECs, TCF7^+^ T cells and CD62L^+^ T cells in vivo. The frequencies of MPECs, TCF7^+^ T cells and CD62L^+^ T cells in NY-ESO-1-specific CD8^+^ T cells in spleens, DLNs and tumors 16 days after tumor inoculation. (B, C) Durable antitumor effects of anti-PD-1 mAb alone or the combination. Mice that completely eradicated the initial CMS5a-NY-ESO-1 tumors by treatment with anti-PD-1 mAb with or without ZSTK474 were re-challenged with CMS5a-NY-ESO-1 (right hind flank) and parental CMS5a (left hind flank) 70 days after the initial tumor inoculation. Individual tumor growth curves for rechallenged CMS5a-NY-ESO-1 and parental CMS5a (B) and survival curves of the re-challenged mice (C). (D, E) CD8^+^ memory T cell responses in spleens, DLNs and tumors after rechallenge with CMS5a-NY-ESO-1 and parental CMS5a in mice that completely eradicated the initial CMS5a-NY-ESO-1 tumors by treatment with anti-PD-1 mAb with/without ZSTK474 or anti-CD25 mAb. The frequencies of NY-ESO-1 (D) or internal tumor antigen in CMS5a (CMS5a-intAg) (E) specific CD8^+^ T cells in spleens, DLNs and tumors after 52 days from the primary tumor inoculation. (F) Effect of anti-CD25 mAb (250 µg/dose; day −1, 150 µg/dose; day 6) on Tregs, NY-ESO-1-specific CD8^+^ T cells and MPECs. The frequencies of Tregs in CD4^+^ T cells, NY-ESO-1-specific CD8^+^ T cells in CD8^+^ T cells and MPECs in NY-ESO-1-specific CD8^+^ T cells in spleens, DLNs and tumors 14 days after tumor inoculation. Data in (A, D–F) are means±SE. Data in (B, C) show pooled data from two independent experiments. Statistical analyses were performed by Dunnett’s test (A, D–F) and Log rank test (C). *P<0.05; **p<0.01; *** p<0.001. DLNs, draining lymph nodes; mAbs, monoclonal antibodies; MPECs, memory precursor effector cells; ns, not significant.

To examine induction of memory T cells by the combination treatment with ZSTK474 and anti-PD-1 mAb, we investigated MPECs in spleens, draining lymph nodes (DLNs) and tumors in mice-bearing CMS5a-NY-ESO-1 tumors after the combination treatment. MPECs in tumor antigen (NY-ESO-1)-specific CD8^+^ T cells in spleens, DLNs and tumors were increased by ZSTK474 treatment alone or in combination with anti-PD-1 mAb (figure 6A and online supplemental figure S6A). Idelalisib treatment also comparably, although slightly less effective, induced MPECs in tumors compared with ZSTK474 either alone or in combination with anti-PD-1 mAb (online supplemental figure S6B). In agreement with the induction of MPECs, ZSTK474 treatment alone or in the combination also increased the memory marker-expressing T cells including TCF7^+^ T cells in tumors and CD62L^+^ T cells in spleens and DLNs although this difference was not statistically significant in spleens (figure 6A and online supplemental figure S6C, D).

10.1136/jitc-2020-002279.supp8 Supplementary data

We then addressed the durability of the antitumor effects based on the enhanced memory T cell generation. Since ZSTK474 treatment alone were not able to completely eradicate the initial CMS5a-NY-ESO-1 tumor, mice that had completely eradicated the initial tumor by treatment with anti-PD-1 mAb with or without ZSTK474 were rechallenged with CMS5a-NY-ESO-1 cells (right hind flank) and parental CMS5a cells (left hind flank) 70 days after primary tumor inoculation. Mice treated with anti-PD-1 mAb alone or combination were resistant to rechallenge with CMS5a-NY-ESO-1 cells in line with NY-ESO-1-specific memory CD8^+^ T cell generation in mice that eradicated the initial tumors, although the frequency of these was low in the anti-PD-1 mAb treatment group (figure 6A, B). Interestingly, parental CMS5a cells in mice treated with the combination were almost completely rejected whereas vigorous growth of parental CMS5a tumors was observed in mice treated with anti-PD-1 mAb alone (figure 6B). Accordingly, the survival rate after rechallenge was significantly improved in the combination treatment group compared with those treated with anti-PD-1 mAb alone (figure 6C).

As CMS5a tumors reportedly harbor an internal tumor antigen (CMS5a-intAg) recognized by CD8^+^ T cells,^31^ we analyzed CD8^+^ T cell responses against NY-ESO-1 and CMS5a-intAg 5 days after the secondary tumor inoculation. NY-ESO-1-specific CD8^+^ T cells were notably enhanced in spleens, DLNs and tumors in the combination treatment group relative to the anti-PD-1 mAb single treatment group (figure 6D). Moreover, CMS5a-intAg-specific CD8^+^ T cells were also more strongly induced in tumors from mice treated with the combination than in those treated with anti-PD-1 mAb alone, although this difference was not statistically significant (figure 6E).

Given that ZSTK474 influenced both Tregs and memory CD8^+^ T cells, we further investigated whether Treg depletion alone was sufficient to induce the durable antitumor effects through increasing MPECs and memory T cell response. To this end, CMS5a-NY-ESO-1-bearing mice were treated with anti-CD25 mAb or anti-CTLA-4 mAb, both of which reportedly induce tumor regression via depleting Tregs,^32^ in combination with anti-PD-1 mAb. Although treatment with anti-CD25 mAb effectively depleted Tregs and increased NY-ESO-1-specific CD8^+^ T cells in combination with anti-PD-1 mAb, this treatment failed to increase MPECs (figure 6F). Similar results were observed with combination of anti-CTLA-4 mAb and anti-PD-1 mAb (online supplemental figure S7A, B). In memory T cell response, mice treated with the combination of anti-CD25 mAb and anti-PD-1 mAb did not manifest any greater induction of NY-ESO-1- or CMS5a-intAg-specific CD8^+^ T cells after inoculation of secondary tumors in any examined tissues than with anti-PD-1 mAb alone (figure 6D, E). Altogether, the combination treatment with ZSTK474 and anti-PD-1 mAb increases memory T cell formation not only against NY-ESO-1 but also an internal tumor antigen(s) present in parental CMS5a cells and augments durable antitumor effects by a mechanism unrelated to Treg reduction and subsequent activation of T cell response via controlling PI3K signaling in CD8^+^ T cells.

10.1136/jitc-2020-002279.supp9 Supplementary data

## Discussion

ICB therapies have shown remarkable clinical benefits in various types of cancer.^1 2^ Yet, clinical benefits are limited to a subset of treated patients.^5 6^ Tregs are involved in the resistance to ICB therapies.^33^ The PI3Kδ inhibitor Idelalisib has been reported to preferentially inhibit Tregs compared with effector T cells.^34^ In the clinical setting, the impairment of Tregs was also observed in peripheral blood from patients with chronic lymphocytic leukemia treated with Idelalisib, although hepatotoxicity characterized by increased infiltrations of T cells was concomitantly observed.^35^ ZSTK474 also induced immune-related toxicities including rash and diarrhea in earlier clinical trials, suggesting augmented immune responses.^36^ Therefore, PI3Kδ inhibitors could be a candidate for combination therapy with ICB therapies. However, Idelalisib was slightly inferior to ZSTK474 in MPEC induction and tumor antigen (NY-ESO-1)-specific CD8^+^ T cell activation. One can then envision that as strong PI3Kδ inhibition impairs activation and proliferation of CD8^+^ T cells and abrogates any advantages of Treg impairment in antitumor immunity,^21^ suggesting that the window of the optimal dose of Idelalisib may be narrow compared with ZSTK474. Another possibility is that PI3Kα and PI3Kβ could compensate for the inhibition of PI3Kδ in Tregs although PI3K signaling in Tregs is mainly dependent on PI3Kδ.^16^ In addition, the timing of PI3K inhibition in ICB therapy may also be a crucial factor because PI3K signaling is required at the early phase of CD8^+^ T cell activation compared with fully activated CD8^+^ T cells.^15^ Therefore, the appropriate timing and dosage of PI3K inhibitor in the combination with ICB needs to be determined to gain the optimal antitumor effects with selective inhibition of Tregs. In this study, we identified the optimal protocol for a PI3K inhibitor ZSTK474 in combination with anti-PD-1 mAb with which a far stronger antitumor immune responses were induced with selective Treg suppression and activation of tumor antigen-specific CD8^+^ T cells. More importantly, the optimized PI3K signaling in CD8^+^ T cells led to the expansion of tumor antigen-specific memory CD8^+^ T cells. The broad memory T cell induction by this combination treatment provides a rationale for developing the combination cancer immunotherapy.

Anti-CTLA-4 mAb (ipilimumab) leads to depletion of Tregs expressing CTLA-4 and yields higher response rates in combination with anti-PD-1 mAb (nivolumab) in patients with some cancer types.^25^ In this combination therapy, nivolumab followed by ipilimumab appears to be more clinically beneficial compared with the opposite order.^26^ In this study, antitumor effects of three different protocols of the combination treatment with ZSTK474 and anti-PD-1 mAb were explored: (1) ZSTK474 and anti-PD-1 mAb were concurrently administered, (2) ZSTK474 single treatment first followed by anti-PD-1 mAb plus ZSTK474 and (3) anti-PD-1 mAb alone first followed by ZSTK474 plus anti-PD-1 mAb. One combination protocol (#3) with anti-PD-1 mAb administrated first, followed by anti-PD-1 mAb plus ZSTKK474 induced effective and durable antitumor activity. Tregs were reduced in the optimal protocol, but not in other protocols. Adding anti-PD-1 mAb to ZSTK474 impaired the reduction of Tregs in those protocols. Anti-PD-1 mAb reportedly activates TCR and CD28 signaling in Tregs, resulting in increasing Treg proliferation and suppressive function.^37^ Therefore, the timing of activation and inhibition of TCR/PI3K signaling by anti-PD-1 mAb and ZSTK474, respectively, in Tregs may be important to obtain the effective and durable antitumor activity by the combination.

The potential usefulness of a combination of Treg depletion with ICB was implied by various preclinical and clinical studies.^33 38^ We have previously shown that Treg depletion using anti-CD25 mAb and anti-CCR4 mAb results in significant activation of effector CD4^+^ cells and CD8^+^ T cells in humans.^39 40^ Yet, as depleting all Tregs can trigger autoimmunity in animal models,^41^ a current key issue is how tumor-infiltrating Tregs can be selectively controlled to evoke and augment antitumor immunity without affecting effector T cells or eliciting deleterious autoimmunity.^39^ Since tumor-infiltrating Tregs are activated via the stimulation of TCR and different costimulatory signals than peripheral Tregs,^7 8 42^ a PI3K inhibitor like ZSTK474 that controls T cell activation signaling could selectively inhibit tumor-infiltrating Tregs, but not peripheral Tregs. This would be an optimal Treg controller in cancer immunotherapy.

A durable clinical effect is one of the most significant characteristics of cancer immunotherapy. Memory CD8^+^ T cell generation is therefore an important goal of current efforts for successful cancer immunotherapies.^43^ ZSTK474 decreased the expression of phosphorylated Akt and S6 not only in Tregs but also in CD8^+^ T cells, although CD8^+^ T cell proliferation was only marginally suppressed by ZSTK474 both in humans and in murine models. This resulted in decreased Tregs but increased CD8^+^ memory T cells, and was associated with prolonged survival of tumor-bearing mice in combination with anti-PD-1 mAb. These opposing outcomes between Tregs and CD8^+^ T cells may be due to inactivation of PI3K signaling by ZSTK474 treatment leaving weak-to-intermediate strength signals that are sufficient to allow CD8^+^ T cell proliferation and differentiation, but not enough for Treg survival. In contrast, other Treg-depleting reagents such as anti-CD25 mAb generally impair both Tregs and CD8^+^ T cells, particularly activated ones, because most molecules including CD25 targeted by the current Treg depletion reagents are concomitantly expressed by activated effector T cells as well.^7 40^ Thus, addition of PI3K inhibitors may result in better clinical responses than other Treg-targeted reagents when combined with PD-1 blockade.

It was thought that memory T cell development requires an intermediate-to-high overall signal strength whereas signals that are too weak or too strong, are not able to support survival or lead to memory T cell generation, and result in only a few memory T cells.^19 29 44 45^ Memory T cell generation for CMS5a-intAg-specific CD8^+^ T cells with low-affinity TCRs, as well as NY-ESO-1-specific CD8^+^ T cells with high-affinity TCRs, was superior in mice treated with a combination of anti-PD-1 mAb and ZSTK474 compared with mice treated with anti-PD-1 mAb alone. Accordingly, mice treated with a combination of anti-PD-1 mAb with ZSTK474 became resistant to rechallenge with CMS5a-NY-ESO-1 cells or CMS5a cells, but mice treated with anti-PD-1 mAb alone were resistant only to CMS5a-NY-ESO-1 re-challenge. One reason for the limited activity of anti-PD-1 mAb alone may be that CMS5a-intAg-specific CD8^+^ T cells are relatively less sensitive to anti-PD-1 mAb than NY-ESO-1-specific CD8^+^ T cells, as PD-1 expression on the former is only weakly induced by CMS5a-intAg-derived TCR signals.^22^ While it is currently unclear why ZSTK474 enhanced memory T cell generation in both high-affinity and low-affinity CD8^+^ T cells, one plausible explanation is that control of PI3K signaling by ZSTK474 may provide a suitable strength of TCR, costimulatory and cytokine signals for memory T cell generation in both high-affinity and low-affinity CD8^+^ T cells. IL-2 and IL-12 signaling reportedly stimulate PI3K signaling and promote terminal differentiation into effector T cells accompanied by the decay of memory T cells.^29^ ZSTK474 may optimize PI3K signaling activated by cytokines including IL-2 and IL-12 in addition to TCR signals in low-affinity CMS5a-intAg-specific CD8^+^ T cells. Another possibility is that high-affinity and low-affinity CD8^+^ T cells have a different threshold for memory T cell generation, and ZSTK474 fine-tunes signal strength preferable for memory T cell generation. Memory T cell generation is controlled by multiple signals including metabolic signals in addition to TCR, costimulatory receptors, cytokines and chemokines.^46^ The glycolysis is enhanced in activated T cells and the oxidative phosphorylation is predominantly used in naive and memory T cells.^47^ PI3K signaling activated by TCR stimulation triggers the recruitment of Glut1 from the cytoplasmic pool to cell surface. Increased Glut1 expression and glucose uptake by activated T cells is accompanied with increased glycolysis.^48^ Thus, ZSTK474 may provide enough energy for the activation of T cells, resulting in the enhanced differentiation into memory T cells via smooth shifting of energy metabolism from glycolytic to oxidative phosphorylation pathways.

The combination of ZSTK474 and anti-PD-1 mAb was well tolerated in our murine models, there were no clinical signs of toxicity, such as body weight loss or mortalities from either of the single agents or the combination. In fact, ZSTK474 doses (100 mg/kg) that were sufficient to deplete Tregs and generate memory CD8^+^ T cells were much lower than those (400 mg/kg) aiming at directly killing cancer cells.^49^ We have recently reported that tyrosine kinase inhibitors such as imatinib also deplete Tregs through targeting TCR signaling.^50^ Importantly, as appropriate T cell activation was achieved in lower doses in both the PI3K inhibitor ZSTK474 and tyrosine kinase inhibitors than the maximum tolerated dose, the dosage used as anticancer reagents may not be optimal for ‘cancer immunotherapy reagent’. It is necessary to select the optimal dose of reagents targeting cellular signaling including ZSTK474 as ‘cancer immunotherapy reagent’. One can then envision that the treatment protocol of reagents that can target both cancer cells and immune cells may be changed during treatment phases based on the purpose of each treatment phase: killing cancer cells versus activating the immune system.

In summary, we develop an optimized combination treatment protocol with a PI3K inhibitor ZSTK474 and PD-1 blockade via selectively inhibiting Tregs and generating memory CD8^+^ T cells, resulting in durable antitumor immunity. This preclinical study provides a rationale for developing the cancer immunotherapies by combining PD-1 blockade with reagents targeting T cell activation signals including PI3K inhibitors. When reagents that target T cell activation signals are employed, optimizing the treatment protocol is essential for maximizing antitumor efficacy of the combination cancer immunotherapy.

## Data Availability

Data are available on reasonable request. The datasets generated and analyzed during the current study are not publicly available due to their relevance only for the study presented here but are available from the corresponding author on reasonable request.

## References

[1] Borghaei H, Paz-Ares L, Horn L, et al. Nivolumab versus docetaxel in advanced Nonsquamous non-small-cell lung cancer. N Engl J Med 2015;373:1627–39. 10.1056/NEJMoa1507643 26412456 PMC5705936

[2] Hodi FS, O'Day SJ, McDermott DF, et al. Improved survival with ipilimumab in patients with metastatic melanoma. N Engl J Med 2010;363:711–23. 10.1056/NEJMoa1003466 20525992 PMC3549297

[3] Schreiber RD, Old LJ, Smyth MJ. Cancer immunoediting: integrating immunity's roles in cancer suppression and promotion. Science 2011;331:1565–70. 10.1126/science.1203486 21436444

[4] Burnet FM. Immunological aspects of malignant disease. Lancet 1967;1:1171–4. 10.1016/S0140-6736(67)92837-1 4165129

[5] Topalian SL, Drake CG, Pardoll DM. Immune checkpoint blockade: a common denominator approach to cancer therapy. Cancer Cell 2015;27:450–61. 10.1016/j.ccell.2015.03.001 25858804 PMC4400238

[6] Lesokhin AM, Callahan MK, Postow MA, et al. On being less tolerant: enhanced cancer immunosurveillance enabled by targeting checkpoints and agonists of T cell activation. Sci Transl Med 2015;7:280sr1. 10.1126/scitranslmed.3010274 25810313

[7] Nishikawa H, Sakaguchi S. Regulatory T cells in cancer immunotherapy. Curr Opin Immunol 2014;27:1–7. 10.1016/j.coi.2013.12.005 24413387

[8] Sakaguchi S, Miyara M, Costantino CM, et al. FOXP3^+^ regulatory T cells in the human immune system. Nat Rev Immunol 2010;10:490–500. 10.1038/nri2785 20559327

[9] Sato E, Olson SH, Ahn J, et al. Intraepithelial CD8^+^ tumor-infiltrating lymphocytes and a high CD8^+^/regulatory T cell ratio are associated with favorable prognosis in ovarian cancer. Proc Natl Acad Sci U S A 2005;102:18538–43. 10.1073/pnas.0509182102 16344461 PMC1311741

[10] Cantley LC. The phosphoinositide 3-kinase pathway. Science 2002;296:1655–7. 10.1126/science.296.5573.1655 12040186

[11] Engelman JA. Targeting PI3K signalling in cancer: opportunities, challenges and limitations. Nat Rev Cancer 2009;9:550–62. 10.1038/nrc2664 19629070

[12] Yaguchi S-ichi, Fukui Y, Koshimizu I, et al. Antitumor activity of ZSTK474, a new phosphatidylinositol 3-kinase inhibitor. J Natl Cancer Inst 2006;98:545–56. 10.1093/jnci/djj133 16622124

[13] Kong D, Dan S, Yamazaki K, et al. Inhibition profiles of phosphatidylinositol 3-kinase inhibitors against PI3K superfamily and human cancer cell line panel JFCR39. Eur J Cancer 2010;46:1111–21. 10.1016/j.ejca.2010.01.005 20129775

[14] Okkenhaug K, Bilancio A, Emery JL, et al. Phosphoinositide 3-kinase in T cell activation and survival. Biochem Soc Trans 2004;32:332–5. 10.1042/bst0320332 15046602

[15] Ali K, Soond DR, Pineiro R, et al. Inactivation of PI(3)K p110δ breaks regulatory T-cell-mediated immune tolerance to cancer. Nature 2014;510:407–11. 10.1038/nature13444 24919154 PMC4501086

[16] Ahmad S, Abu-Eid R, Shrimali R, et al. Differential PI3Kδ Signaling in CD4^+^ T-cell Subsets Enables Selective Targeting of T Regulatory Cells to Enhance Cancer Immunotherapy. Cancer Res 2017;77:1892–904. 10.1158/0008-5472.CAN-16-1839 28108509

[17] Abu-Eid R, Samara RN, Ozbun L, et al. Selective inhibition of regulatory T cells by targeting the PI3K-Akt pathway. Cancer Immunol Res 2014;2:1080–9. 10.1158/2326-6066.CIR-14-0095 25080445 PMC4221428

[18] Carnevalli LS, Sinclair C, Taylor MA, et al. PI3Kα/δ inhibition promotes anti-tumor immunity through direct enhancement of effector CD8^+^ T-cell activity. J Immunother Cancer 2018;6:158. 10.1186/s40425-018-0457-0 30587236 PMC6307194

[19] Kim EH, Suresh M. Role of PI3K/Akt signaling in memory CD8 T cell differentiation. Front Immunol 2013;4:20. 10.3389/fimmu.2013.00020 23378844 PMC3561661

[20] Kaech SM, Cui W. Transcriptional control of effector and memory CD8^+^ T cell differentiation. Nat Rev Immunol 2012;12:749–61. 10.1038/nri3307 23080391 PMC4137483

[21] Lim EL, Cugliandolo FM, Rosner DR, et al. Phosphoinositide 3-kinase δ inhibition promotes antitumor responses but antagonizes checkpoint inhibitors. JCI Insight 2018;3. 10.1172/jci.insight.120626 PMC6124416 29875319

[22] Tokunaga A, Sugiyama D, Maeda Y, et al. Selective inhibition of low-affinity memory CD8^+^ T cells by corticosteroids. J Exp Med 2019;216:2701–13. 10.1084/jem.20190738 31537643 PMC6888983

[23] Curran MA, Montalvo W, Yagita H, et al. PD-1 and CTLA-4 combination blockade expands infiltrating T cells and reduces regulatory T and myeloid cells within B16 melanoma tumors. Proc Natl Acad Sci U S A 2010;107:4275–80. 10.1073/pnas.0915174107 20160101 PMC2840093

[24] Hudson K, Hancox UJ, Trigwell C, et al. Intermittent high-dose scheduling of AZD8835, a novel selective inhibitor of PI3Kα and PI3Kδ, demonstrates treatment strategies for PIK3CA-Dependent breast cancers. Mol Cancer Ther 2016;15:877–89. 10.1158/1535-7163.MCT-15-0687 26839307

[25] Wolchok JD, Chiarion-Sileni V, Gonzalez R, et al. Overall survival with combined nivolumab and ipilimumab in advanced melanoma. N Engl J Med 2017;377:1345–56. 10.1056/NEJMoa1709684 28889792 PMC5706778

[26] Weber JS, Gibney G, Sullivan RJ, et al. Sequential administration of nivolumab and ipilimumab with a planned switch in patients with advanced melanoma (CheckMate 064): an open-label, randomised, phase 2 trial. Lancet Oncol 2016;17:943–55. 10.1016/S1470-2045(16)30126-7 27269740 PMC5474305

[27] Coulie PG, Van den Eynde BJ, van der Bruggen P, et al. Tumour antigens recognized by T lymphocytes: at the core of cancer immunotherapy. Nat Rev Cancer 2014;14:135–46. 10.1038/nrc3670 24457417

[28] Lin W-HW, Nish SA, Yen B, et al. CD8^+^ T Lymphocyte Self-Renewal during Effector Cell Determination. Cell Rep 2016;17:1773–82. 10.1016/j.celrep.2016.10.032 27829149 PMC5108530

[29] Joshi NS, Cui W, Chandele A, et al. Inflammation directs memory precursor and short-lived effector CD8^+^ T cell fates via the graded expression of T-bet transcription factor. Immunity 2007;27:281–95. 10.1016/j.immuni.2007.07.010 17723218 PMC2034442

[30] Youngblood B, Hale JS, Kissick HT, et al. Effector CD8 T cells dedifferentiate into long-lived memory cells. Nature 2017;552:404–9. 10.1038/nature25144 29236683 PMC5965677

[31] Ikeda H, Ohta N, Furukawa K, et al. Mutated mitogen-activated protein kinase: a tumor rejection antigen of mouse sarcoma. Proc Natl Acad Sci U S A 1997;94:6375–9. 10.1073/pnas.94.12.6375 9177225 PMC21057

[32] Togashi Y, Shitara K, Nishikawa H. Regulatory T cells in cancer immunosuppression - implications for anticancer therapy. Nat Rev Clin Oncol 2019;16:356–71. 10.1038/s41571-019-0175-7 30705439

[33] Pitt JM, Vétizou M, Daillère R, et al. Resistance mechanisms to Immune-Checkpoint blockade in cancer: tumor-intrinsic and -extrinsic factors. Immunity 2016;44:1255–69. 10.1016/j.immuni.2016.06.001 27332730

[34] Chellappa S, Kushekhar K, Munthe LA, et al. The PI3K p110δ isoform inhibitor idelalisib preferentially inhibits human regulatory T cell function. J Immunol 2019;202:1397–405. 10.4049/jimmunol.1701703 30692213

[35] Lampson BL, Kasar SN, Matos TR, et al. Idelalisib given front-line for treatment of chronic lymphocytic leukemia causes frequent immune-mediated hepatotoxicity. Blood 2016;128:195–203. 10.1182/blood-2016-03-707133 27247136 PMC4946200

[36] Lockhart AC, Olszanski AJ, Allgren RL. Abstract B271: A first-in-human Phase I study of ZSTK474, an oral pan-PI3K inhibitor, in patients with advanced solid malignancies. Molecular Cancer Therapeutics 2013;12:B271. 10.1158/1535-7163.TARG-13-B271

[37] Kumagai S, Togashi Y, Kamada T, et al. The PD-1 expression balance between effector and regulatory T cells predicts the clinical efficacy of PD-1 blockade therapies. Nat Immunol 2020;21:1346–58. 10.1038/s41590-020-0769-3 32868929

[38] Arce Vargas F, Furness AJS, Solomon I, et al. Fc-Optimized Anti-CD25 depletes tumor-infiltrating regulatory T cells and synergizes with PD-1 blockade to eradicate established tumors. Immunity 2017;46:577–86. 10.1016/j.immuni.2017.03.013 28410988 PMC5437702

[39] Shimizu J, Yamazaki S, Sakaguchi S. Induction of tumor immunity by removing CD25^+^CD4^+^ T cells: a common basis between tumor immunity and autoimmunity. J Immunol 1999;163:5211–8. 10553041

[40] Sugiyama D, Nishikawa H, Maeda Y, et al. Anti-CCR4 mAb selectively depletes effector-type FoxP3^+^CD4^+^ regulatory T cells, evoking antitumor immune responses in humans. Proc Natl Acad Sci U S A 2013;110:17945–50. 10.1073/pnas.1316796110 24127572 PMC3816454

[41] Sakaguchi S. Naturally arising CD4^+^ regulatory T cells for immunologic self-tolerance and negative control of immune responses. Annu Rev Immunol 2004;22:531–62. 10.1146/annurev.immunol.21.120601.141122 15032588

[42] Nishikawa H, Kato T, Tawara I, et al. Definition of target antigens for naturally occurring CD4^+^ CD25^+^ regulatory T cells. J Exp Med 2005;201:681–6. 10.1084/jem.20041959 15753203 PMC2212825

[43] Reading JL, Gálvez-Cancino F, Swanton C, et al. The function and dysfunction of memory CD8^+^ T cells in tumor immunity. Immunol Rev 2018;283:194–212. 10.1111/imr.12657 29664561

[44] Teixeiro E, Daniels MA, Hamilton SE, et al. Different T cell receptor signals determine CD8^+^ memory versus effector development. Science 2009;323:502–5. 10.1126/science.1163612 19164748

[45] Zehn D, Lee SY, Bevan MJ. Complete but curtailed T-cell response to very low-affinity antigen. Nature 2009;458:211–4. 10.1038/nature07657 19182777 PMC2735344

[46] Chang JT, Wherry EJ, Goldrath AW. Molecular regulation of effector and memory T cell differentiation. Nat Immunol 2014;15:1104–15. 10.1038/ni.3031 25396352 PMC4386685

[47] Rangel Rivera GO, Knochelmann HM, Dwyer CJ, et al. Fundamentals of T cell metabolism and strategies to enhance cancer immunotherapy. Front Immunol 2021;12:645242. 10.3389/fimmu.2021.645242 33815400 PMC8014042

[48] Palmer CS, Ostrowski M, Balderson B, et al. Glucose metabolism regulates T cell activation, differentiation, and functions. Front Immunol 2015;6:1. 10.3389/fimmu.2015.00001 25657648 PMC4302982

[49] Dan S, Okamura M, Seki M, et al. Correlating phosphatidylinositol 3-kinase inhibitor efficacy with signaling pathway status: in silico and biological evaluations. Cancer Res 2010;70:4982–94. 10.1158/0008-5472.CAN-09-4172 20530683

[50] Tanaka A, Nishikawa H, Noguchi S, et al. Tyrosine kinase inhibitor imatinib augments tumor immunity by depleting effector regulatory T cells. J Exp Med 2020;217. 10.1084/jem.20191009. [Epub ahead of print: 03 02 2020]. PMC7041710 31704808

