# Supplementary material for: Cancer immunotherapy with PI3K and PD-1 dual-blockade via optimal modulation of T cell activation signal
Source: J Immunother Cancer. 2021 Aug 20;9(8):e002279. doi: 10.1136/jitc-2020-002279 (PMC8395371; doi:10.1136/jitc-2020-002279)
Supplement: Supplementary data [file jitc-2020-002279supp002.pdf]

Supplemental Materials and Methods

In vivo animal model

Female BALB/c or C57BL/6 mice were inoculated subcutaneously with 2×10^6 CMS5a-NY-ESO-1 cells or 1×10^6 B16F0 cells, respectively, in the right hind flank (day 0). When the tumors reached 4-6 mm in diameter, mice were randomized into ZSTK474-treated, Idelalisib-treated, anti-CD25 mAb-treated, anti-CTLA4 mAb-treated or anti-PD-1 mAb-treated groups. Oral administration was once a day with ZSTK474 (30, 100 or 300 mg/kg suspended in 5% hydroxypropylmethylcellulose in water) or Idelalisib (100 mg/kg) and intraperitoneal injection twice a week with anti-CD25 mAb (250 µg/dose or 150 µg/dose), anti-CTLA4 mAb (200 µg/dose) or anti-PD-1 mAb (250 µg/dose).

Upon oral administration of 30, 100 and 300 mg/kg ZSTK474 to BALB/c mice, the mean ZSTK474 area under the plasma concentration time curve from 0 to 24 h (AUC_{0-24}) values were 5392.7±1442.0, 13157.8±1925.1 and 29040.7±1544.1 h*ng/ml, respectively. Following oral administration of the maximum tolerated dose (MTD: 150 mg) to patients with solid tumor, the mean of total ZSTK474 and active metabolite AUC_{0-24} value is 11545.5±6539.8 h*ng/ml.[1] One of the major active metabolites of ZSTK474 detected in human peripheral blood shows 5-fold higher activity to inhibit PI3Kδ than ZSTK474. ZSTK474 also inhibits human PI3Kδ with a 3-6-fold higher activity over murine PI3Kδ,[2] indicating that doses (30 - 300 mg/kg) of ZSTK474 to mice tested in this study are comparable to the ranges of clinical exposure doses of MTD in humans.

In the secondary challenge experiments, tumor-free mice in each treatment group were re-challenged subcutaneously with 2×10^7 CMS5a-NY-ESO-1 (right hind flank) and 2×10^6 parental CMS5a cells (left hind flank).

Isoyama S, *et al. J Immunother Cancer* 2022; 9:e002279. doi: 10.1136/jitc-2020-002279

For *in vivo* cell depletion, anti-CD4 mAb (GK1.5, 500 μg/dose) or anti-CD8β mAb (53-5.8, 500 μg/dose) was administered one day before ZSTK474, and each mAb repeatedly injected (250 μg/dose) seven days after the previous administration. Tumor volume (TV) was monitored twice a week by measurement of the length (L) and width (W) of the subcutaneous tumor mass using calipers, and the TV was calculated as TV = $(L \times W^2)/2$.

Murine cell isolation and staining

Spleens, DLNs and tumors were dissociated mechanically into single-cell suspension using a Gentle MACS Dissociator (Miltenyi Biotec, Bergisch Gladbach, Germany) in ice-cold PBS supplemented with 2% FBS or ice-cold PBS supplemented with 0.5% bovine serum albumin (BSA) and 2 mM EDTA. After filtration through nylon mesh, lymphocytes were stained with APC-labeled NY-ESO-1/Dd tetramer or PE-labeled CMS5a-intAg/Kd tetramer (TCMetrix, Epalinges, Switzerland) for 10 min at 37°C. Lymphocytes were further stained with mAbs and fixable viability dye for 15 min at 4°C. Intracellular staining was carried out with a FoxP3/Transcription Factor Staining Buffer Kit according to the manufacturer's instructions (Thermo Fisher Scientific, Waltham, MA). After washing, cells were analyzed on an LSR Fortessa (BD Biosciences), a FACSymphony (BD Biosciences) and FlowJo software (BD Biosciences).

Human samples

Peripheral blood mononuclear cells (PBMCs) from healthy individuals were purchased from Cellular Technology Limited (Cleveland, OH) or Precision For Medicine (Bethesda, MD). PBMCs from gastric cancer patients were obtained from National

Isoyama S, *et al. J Immunother Cancer* 2022; 9:e002279. doi: 10.1136/jitc-2020-002279

Cancer Center Japan Hospital East. All cancer patients provided written informed consent before sampling according to the Declaration of Helsinki. This study was approved by the institutional ethics committees of National Cancer Center Japan and Zenyaku Kogyo, Co., Ltd.

In vitro CD3/CD28 stimulation

For T cell proliferation assays, $CD4^{+}CD25^{high}$, $CD4^{+}CD25^{-}$ and $CD8^{+}$ T cells were sorted from human PBMCs with a FACSAria Fusion (BD Biosciences). The purity of each T cell fraction was confirmed to be >95%. 5 x 10^{5} CFSE (Thermo Fisher Scientific)-labelled cells were cultured with anti-CD3/CD28-conjugated Dynabeads (Life Technologies, Carlsbad, CA) at a 1:1 cell-to-bead ratio in RPMI-1640 supplemented with 10% human AB serum (Gemini Bio-Products, Calabasas, CA) and 4 mmol/L of L-glutamine for 4 days in the presence or absence of titrated doses of ZSTK474. CFSE dilution was then assessed by flow cytometry. For human memory T cell induction assays, $CD8^{+}$ T cells prepared from human PBMCs by negative selection with a $CD8^{+}$ isolation kit (Miltenyi Biotec) were further separated into naive $CD8^{+}$ T cells ($CD45RA^{+}CCR7^{+}$) and effctor $CD8^{+}$ T cells ($CD45RA^{-}CCR7^{-}$) with a FACSAria Fusion (BD Biosciences). The purity was confirmed to be >95%. Sorted naive $CD8^{+}$ T cells and effector $CD8^{+}$ T cells were cultured in 24-well plates pre-coated with anti-CD3 mAb (1 μg/ml) and anti-CD28 mAb (1 μg/ml) for 8 days in the presence or absence of ZSTK474.

In vitro sensitization of antigen-specific $CD8^{+}$ T cells

1.5-2.0 × 10^{6} human PBMCs were cultured with 10 μM CMV (NLVPMVATV), Flu (GILGFVFTL) or Melan-A (ELAGIGILTV) peptide in round-bottom 96-well plates

with or without ZSTK474. After 8 h, one-half of the medium was replaced by fresh medium containing IL-2 (20 U/mL) and IL-7 (40 ng/mL, R&D Systems, Minneapolis, MN) with or without ZSTK474, and repeated twice per week. In other experiments using PBMCs from cancer patients or healthy individuals, $CD8^+$ T cells were isolated from PBMCs by positive selection on a MACS column using anti-CD8 microbeads (Miltenyi Biotec). Non-$CD8^+$ cells were separated into $CD4^+$ cells and $CD8^-CD4^-$ cells with anti-CD4 microbeads (Miltenyi Biotec). $CD8^-CD4^-$ cells were pulsed with 10 μM of peptide antigen overnight to be used as APC. After irradiation, 1-5 × 10^5 APCs were added to round-bottom 96-well plates containing 0.5-3 × 10^5 $CD8^+$ T cells in the presence or absence of ZSTK474. Subsequently, one-half of the medium was replaced by fresh medium containing IL-2 (20 U/ml) and IL-7 (40 ng/mL) with or without ZSTK474 twice per week.

Human cell staining

Cells were stained with PE-labeled CMV/HLA-A*0201 tetramer, PE-labeled Flu/HLA-A*0201 tetramer, PE-labeled Melan-A/HLA-A*0201 tetramer, PE-eFluor 610 labeled WT-1/HLA-A*0201 tetramer, PE-labeled NY-ESO-1/HLA-A*0201 tetramer, PE-labeled MAGE-A3/HLA-A*0201 tetramer or APC-labeled MAGE-A4/HLA-A*2402 tetramer (TCMetrix, Epalinges, Switzerland) for 10 min at 37°C. Cells were further stained with mAbs and fixable viability dye for 15 min at 4°C. Intracellular staining was carried out using a FoxP3/Transcription Factor Staining Buffer Kit according to the manufacturer's instructions (Thermo Fisher Scientific). Permeabilized and fixed cells were stained with mAbs for 15 min at 4°C. After washing, cells were analyzed with an LSR Fortessa (BD Biosciences), a FACSymphony (BD Biosciences) and FlowJo software (BD Biosciences).

Isoyama S, *et al. J Immunother Cancer* 2022; 9:e002279. doi: 10.1136/jitc-2020-002279

Phospho-flow cytometry

Mice bearing CMS5a-NY-ESO-1 were orally administrated with ZSTK474 at 30, 100 or 300 mg/kg once a day for 7 days. After 2 hours from the last administration of ZSTK474, DLNs were collected from the mice. DLNs were dissociated mechanically into single-cell suspension in ice-cold PBS supplemented with 0.5% bovine serum albumin (BSA) and 2 mM EDTA. After filtration through nylon mesh, lymphocytes were stained for surface proteins. After washing, the stained cells were fixed with BD Cytofix Fixation Buffer (BD Biosciences). The fixed cells were permeabilized with BD Phosflow Perm Buffer I or III (BD Biosciences) followed by FoxP3/Transcription Factor Staining Buffer Kit (Thermo Fisher Scientific) and stained for intracellular proteins including phosphorylated proteins and FOXP3. The flow cytometry data were analyzed with a FACSymphony (BD Biosciences). Human PBMCs were incubated with indicated concentrations of ZSTK474 for 30 min on ice and stimulated at the indicated time in each figure at 37°C with anti-CD3 mAb (1 μg/ml), anti-CD28 mAb (1 μg/ml) and anti-mouse Ig polyclonal antibody (20 μg/ml). After stimulation, the cells were put on ice and stained for surface proteins. The protocols after that is the same as mice lymphocytes described above.

Immunoblotting

Purified T cells as indicated were stimulated with anti-CD3/CD28-conjugated Dynabeads for 30 min at 37°C with or without ZSTK474. Immunoblot assays were carried out on cell extracts using primary antibody against phosphorylated ribosomal S6 protein at Ser235/236 and β-actin followed by horseradish peroxidase-linked secondary antibody as described previously.[3] Images were acquired using a LAS 4000 (Fujifilm,

Isoyama S, et al. J Immunother Cancer 2022; 9:e002279. doi: 10.1136/jitc-2020-002279

Tokyo, Japan) and quantification of bands was carried out using Multi Gauge Ver3.1 (Fujifilm).

RNA-seq analysis

Purified naive $CD8^+$ ($CD45RA^+CCR7^+$) T cells were stimulated with plate-bound anti-CD3 mAb and anti-CD28 mAb for 24 or 48 hours at 37°C with or without ZSTK474. Those cells were lysed in TRIzol (Thermo Fisher), followed by RNA extraction according to the manufacturer's instructions. The isolated RNA was purified using RNeasy Plus Mini Kits (Qiagen, Hilden, Germany). After a quality control step with a 2100 bioanalyzer (Agilent Technologies, Palo Alto, CA), library was constructed using a TruSeq library protocol, and subjected to deep sequencing with an Illumina sequencer HiSeq2500. In order to remove technical sequences from raw fastq data, including adapters, polymerase chain reaction (PCR) primers, or fragments thereof, and quality of bases lower than 20, pass filter data of fastq format were processed by Cutadapt (V1.9.1) to be high quality clean data. The clean data were aligned to UCSC hg38 reference genome via software Hisat2 (v2.0.1). Then, with the file as a reference gene file, HTSeq (v0.6.1) estimated gene and isoform expression levels from the pair-end clean data. Principal component and cluster analyses were performed using genefilter package in R. GSEA was performed using GSEA v4.0.3 software. Naive, effector and memory $CD8^+$ T cell signatures were downloaded from MSigDB: Goldrath naive vs eff and eff vs memory $CD8^+$ T cell,[4] Kaech naive vs Day8 eff and Day8 eff vs memory $CD8^+$ T cell,[5] GSE10239 naive vs Day4.5 eff and memory vs Day4.5 eff $CD8^+$ T cell.[6] Activated T cell gene set was from Singer, M. et. al.[7]-supplementary table 4 and $TCF7^+$ or $TCF7^-$ TILs gene set was generated from Siddiqui, I. et. al.[8]-Table S1.

Quantitative real-time PCR

Purified naive $CD8^+$ ($CD45RA^+CCR7^+$) T cells were stimulated with plate-bound anti-CD3 mAb and anti-CD28 mAb for 24 or 48 hours at 37°C with or without ZSTK474. RNA was extracted using RNeasy Plus Mini Kits (Qiagen, Hilden, Germany) and cDNA was synthesized using SuperScript VILO Master Mix (Thermo Fisher Scientific). Quantitative real-time PCR was performed with SYBR Green (Roche Diagnostics, Basel, Switzerland) and LightCycler (Roche) according to the manufacturer's instructions. Primers for *TCF-7*, *BCL-6*, *EOMES*, *T-BET* and *IL7R* were purchased from Thermo Fisher Scientific. *ACTB* was used as an internal control.

Protein kinase inhibition assay

To examine selectivity of kinase inhibition of ZSTK474, 261 protein kinases were tested. Kinase inhibition was measured using radiometric assays performed by KinaseProfiler service (Millipore) with 1 µM of ZSTK474.

References

1. Lockhart AC, Olszanski AJ, Allgren RL, Yaguchi S, Cohen SJ, Hilton JF, Wang-Gillam A, Shapiro GI. Abstract B271: A first-in-human Phase I study of ZSTK474, an oral pan-PI3K inhibitor, in patients with advanced solid malignancies. Molecular Cancer Therapeutics 2013;12:B271

2. Kong D, Dan S, Yamazaki K, Yamori T. Inhibition profiles of phosphatidylinositol 3-kinase inhibitors against PI3K superfamily and human cancer cell line panel JFCR39. Eur J Cancer 2010;46:1111-21

3. Isoyama S, Dan S, Nishimura Y, Nakamura N, Kajiwara G, Seki M*, et al.*

Establishment of phosphatidylinositol 3-kinase inhibitor-resistant cancer cell lines and therapeutic strategies for overcoming the resistance. Cancer Sci **2012**;103:1955-60

4. Luckey CJ, Bhattacharya D, Goldrath AW, Weissman IL, Benoist C, Mathis D. Memory T and memory B cells share a transcriptional program of self-renewal with long-term hematopoietic stem cells. Proc Natl Acad Sci U S A **2006**;103:3304-9

5. Kaech SM, Hemby S, Kersh E, Ahmed R. Molecular and functional profiling of memory CD8 T cell differentiation. Cell **2002**;111:837-51

6. Sarkar S, Kalia V, Haining WN, Konieczny BT, Subramaniam S, Ahmed R. Functional and genomic profiling of effector CD8 T cell subsets with distinct memory fates. J Exp Med **2008**;205:625-40

7. Singer M, Wang C, Cong L, Marjanovic ND, Kowalczyk MS, Zhang H, *et al.* A Distinct Gene Module for Dysfunction Uncoupled from Activation in Tumor-Infiltrating T Cells. Cell **2016**;166:1500-11.e9

8. Siddiqui I, Schaeuble K, Chennupati V, Fuertes Marraco SA, Calderon-Copete S, Pais Ferreira D, *et al.* Intratumoral $Tcf1^{+}PD\text{-}1^{+}CD8^{+}$ T Cells with Stem-like Properties Promote Tumor Control in Response to Vaccination and Checkpoint Blockade I mmunotherapy. Immunity **2019**;50:195-211.e10

Figure legends

Figure 1. Treatment of ZSTK474 alone or in combination with anti-PD-1 mAb activates antitumor immunity via Treg suppression.

A. The relative changes of intra-tumoral Treg, tumor antigen-specific CD8$^+$ T cell, CD4$^+$ T cell and CD8$^+$ T cell counts per tumor volume (mm^3) by ZSTK474 treatment in mice bearing CMS5a-NY-ESO-1. ZSTK474 was administrated at 30, 100 or 300 mg/kg once a day from day 6 to 13. T cells were collected from tumors 14 days after tumor inoculation and were subjected to flow cytometry. Data are means ±SD. **B.** Phosphorylation status of Akt at S473 (p-Akt) and S6 at S235/236 (p-S6) in Tregs, helper CD4$^+$ T cells and CD8$^+$ T cells in DLNs of mice bearing CMS5a-NY-ESO-1 treated with ZSTK474 at 30, 100 or 300 mg/kg once a day from day 6 to 12. DLNs were collected from mice 2 hours after the last administration and T cells in DLNs were subjected to flow cytometry. Representative flow cytometry histograms (upper panels) and summaries of mean fluorescence (MFI) of p-Akt and p-S6 in Tregs, helper CD4$^+$ T cells and CD8$^+$ T cells are shown. **C.** Tumor growth inhibition of CMS5a-NY-ESO-1 tumors by ZSTK474 treatment (n = 6 per group) in CD4$^+$ or CD8$^+$ T cell-deleted mice. **D.** Antitumor effects of the combination therapy with ZSTK474 and anti-PD-1 mAb (three different protocols) in CMS5a-NY-ESO-1 model (n = 8 per group). ZSTK474 and anti-PD-1 mAb were administered as shown in each upper panel. **E.** Survival curves of CMS5a-NY-ESO-1-bearing mice treated with or without ZSTK474 (once a day from day 9 to 28) and/or anti-PD-1 mAb (day 6, 9, 13 and 16). **F, G.** Mice bearing CMS5a-NY-ESO-1 were treated with or without ZSTK474 (once a day from day 9 to 15) and/or anti-PD-1 mAb (day 6, 9 and 13). T cells were collected from spleens and tumors at 16 days after tumor inoculation and were subjected to flow cytometry. The frequencies of Tregs in CD4$^+$ T cells in spleens (left) and tumors (right in **F**), the frequencies of NY-

ESO-1 specific $CD8^+$ T cells (left), $CD8^+$ T cell:Treg ratio (middle) and NY-ESO-1 specific $CD8^+$ T cell:Treg ratio (right in **G**) in tumors. Data are means ±SE. Statistical analyses were performed by Dunnett's test (**A, B, D, F, G**), Student's *t* test (**C**) and Gehan-Breslow-Wilcoxon test (**E**). These experiments were performed independently at least two to three times with similar results. *, $P < 0.05$; **, $P < 0.01$; ***, $P<0.001$.

Isoyama S, *et al. J Immunother Cancer* 2022; 9:e002279. doi: 10.1136/jitc-2020-002279

Figure 2. Inhibition of PI3K signaling by ZSTK474 increases induction of antigen-specific CD8$^+$ T cells by selectively inhibiting Tregs in humans.

A-C. CD8$^+$ T cell responses to CMV (**A**), Flu (**B**) or Melan-A (**C**) peptides treated with the indicated doses of ZSTK474. Antigen-specific CD8$^+$ T cells in peripheral blood mononuclear cells (PBMCs) from healthy individuals were detected by MHC/peptide multimers. Representative flow cytometric analysis (left) and summaries of triplicate data (right). The numbers in the panels indicate the frequencies of gated CD8$^+$ T cells. These experiments were performed independently at least two to three times with similar results. **D, E.** Tregs (CD4$^+$CD25^{high}), helper CD4$^+$ T cells (CD4$^+$CD25$^-$) and CD8$^+$ T cells were prepared from human PBMCs and labelled with CFSE. Proliferation was examined by CFSE dilution after stimulation with anti-CD3/anti-CD28 mAb for four days with or without the indicated dose of ZSTK474 or Idelalisib. A representative staining (left) and summaries of three independent experiments (right). The numbers in the panels indicate the frequencies of proliferated T cells. Data are means ±SE. Statistical analyses were performed by Dunnett's test (**A**-**C**) and Tukey's test (**D, E**). *, $P < 0.05$; **, $P < 0.01$; ***, $P<0.001$.

Figure 3. ZSTK474 inhibits PI3K signaling not only in Tregs but also in CD8^{+} T cells.

A, B. Phosphorylation status of SLP-76 at Y128 (p-SLP-76) and Akt at S473 (p-Akt) after stimulation with anti-CD3/anti-CD28 mAb at the indicated time points with the indicated doses of ZSTK474. Representative flow cytometry histograms (**A**) and summaries of mean fluorescence (MFI) of p-SLP-76 and p-Akt (**B**) in Tregs, helper CD4^{+} T cells and CD8^{+} T cells. Data are means ±SE. **C.** Phosphorylation of Akt at S473 (p-Akt) and S6 at S235/236 (p-S6) after stimulation with anti-CD3/anti-CD28 mAb for 20 min with or without ZSTK474 (1 or 10 μM). Representative pictures of immunoblot analyses (left) and the average of quantification values of bands (right). Data are means ±SD. Statistical analyses were performed by Student's *t* test (**B**), and Dunnett's test (**C**). *, $P < 0.05$; **, $P < 0.01$; ***, $P<0.001$.

Isoyama S, *et al. J Immunother Cancer* 2022; 9:e002279. doi: 10.1136/jitc-2020-002279

Figure 4. CD8$^+$ T cells treated with ZSTK474 exhibits memory T cell-like gene expression profiles.

A-G. Naive CD8$^+$ T cells (CD45RA$^+$CCR7$^+$) prepared from PBMCs of three healthy individuals were stimulated with anti-CD3/anti-CD28 mAb for 24 and 48 hours with or without ZSTK474 (1 μM) and subjected to RNA-seq analysis. **A, B.** Principal component analysis (**A**) and unsupervised hierarchical clustering (**B**) of gene expression profiles in unstimulated naive CD8$^+$ T cells and CD8$^+$ T cells stimulated for 24 or 48 hours. **C, D.** Representative Gene-set enrichment analysis (GSEA) plots (**C**) and summaries of GSEA (**D**). The enrichment of naive or effector CD8$^+$ T cell signatures in unstimulated CD8$^+$ T cells versus stimulated CD8$^+$ T cells is shown. Color scale represents the enrichment score in unstimulated CD8$^+$ T cells compared to that in stimulated CD8$^+$ T cells. Circle size indicates the False Discovery Rate (FDR) in (**D**). **E, F.** Representative GSEA plots (**E**) and summaries of GSEA (**F**). The enrichment of effector or memory CD8$^+$ T cell signatures in stimulated CD8$^+$ T cells without versus with ZSTK474 is shown. **G.** Heatmap showing relative gene expression of effector and memory T cell-associated genes in CD8$^+$ T cells stimulated with vs without ZSTK474 for 24 or 48 hours. **H.** Real-time quantitative RT-PCR analysis of memory T cell-associated transcription factors. mRNA levels of *TCF-7*, *BCL-6*, *EOMES*, *T-BET* and *IL7R* were measured. Data are the average of triplicate assays and are means ±SE. These experiments were performed independently at least twice to three times with similar results. **I.** Schematic illustration of Waddington's landscape model showing differentiation of naive CD8$^+$ T cells into effector and memory T cells by antigen stimulation in the presence or absence of ZSTK474, a PI3K inhibitor. Statistical analyses were performed by Student's *t* test (**H**). *, $P < 0.05$; **, $P < 0.01$.; ***, $P<0.001$.

Isoyama S, *et al. J Immunother Cancer* 2022; 9:e002279. doi: 10.1136/jitc-2020-002279

Figure 5. Inhibition of PI3K signaling by ZSTK474 in CD8$^+$ T cells enhances memory T cell differentiation.

A-F. Naive CD8$^+$ T cells (CD45RA$^+$CCR7$^+$) (**A-E**) and effector CD8$^+$ T cells (CD45RA$^-$CCR7$^-$) (**F**) prepared from PBMCs of healthy individuals were stimulated with anti-CD3/anti-CD28 mAb for eight days with or without the indicated doses of ZSTK474 or Idelalisib. Representative flow cytometry staining (left) and summary (right) of the frequencies of MPECs (KLRG1$^-$CD127$^+$) in CD8$^+$ T cells (**A**), the frequencies of activated-caspase-3$^+$CD8$^+$ T cells in CD8$^+$ T cells (**B**), the Mean Fluorescence Intensity (MFI) of Ki67 (**C**), summary of the frequencies of MPECs (KLRG1$^-$CD127$^+$) in CD8$^+$ T cells (**D**), absolute counts of MPEC per well in 96-well plates (**E**) and the frequencies of MPECs (KLRG1$^-$CD127$^+$) in CD8$^+$ T cells (**F**). The numbers in the panels indicate the frequencies in CD8$^+$ T cells (**A**, **B**) and the MFI of Ki67 in the indicated cells with the same color (**C**). **G, H.** CD8$^+$ T cells in PBMCs from healthy individuals were stimulated by X-irradiated APCs pulsed with Melan-A or CMV peptide with or without the indicated doses of ZSTK474 for eight days. Representative flow cytometry staining (left) and summaries (right) of the frequencies of antigen-specific CD8$^+$ T cells (**G**) and MPECs in antigen-specific CD8$^+$ T cells (**H**). The numbers in the panels indicate the frequencies in CD8$^+$ T cells (**G**) and the frequencies in antigen-specific CD8$^+$ T cells (**H**). **I, J.** CD8$^+$ T cell responses against cancer antigens treated with the indicated doses of ZSTK474. CD8$^+$ T cells in PBMCs from gastric cancer patients were cultured with or without ZSTK474 in the presence of X-irradiated CD4$^-$CD8$^-$ PBMCs as APCs pulsed with WT-1, NY-ESO-1, MAGE-A3 or MAGE-A4 peptide. Flow cytometry staining of antigen-specific CD8$^+$ T cells (**I**) and MPECs in antigen-specific CD8$^+$ T cells (**J**). The experiments (**A-H**) were performed

Isoyama S, *et al. J Immunother Cancer* 2022; 9:e002279. doi: 10.1136/jitc-2020-002279

independently at least two to three with similar results. Data are means ±SE. Statistical analyses were performed by Dunnett's test (**D**-**H**). **, P < 0.01; ***, P<0.001.

Isoyama S, *et al. J Immunother Cancer* 2022; 9:e002279. doi: 10.1136/jitc-2020-002279

Figure 6. ZSTK474 but not anti-CD25 mAb in combination with anti-PD-1 mAb increases memory T cells *in vivo*, resulting in augmented durable antitumor effects. **A.** Effects of ZSTK474 and anti-PD-1 mAb either alone or in combination on induction of MPECs, $TCF7^+$ T cells and $CD62L^+$ T cells *in vivo*. The frequencies of MPECs, $TCF7^+$ T cells and $CD62L^+$ T cells in NY-ESO-1-specific $CD8^+$ T cells in spleens, DLNs and tumors 16 days after tumor inoculation. **B, C.** Durable antitumor effects of anti-PD-1 mAb alone or the combination. Mice that completely eradicated the initial CMS5a-NY-ESO-1 tumors by treatment with anti-PD-1 mAb with or without ZSTK474 were re-challenged with CMS5a-NY-ESO-1 (right hind flank) and parental CMS5a (left hind flank) 70 days after the initial tumor inoculation. Individual tumor growth curves for re-challenged CMS5a-NY-ESO-1 and parental CMS5a (**B**) and survival curves of the re-challenged mice (**C**). **D, E.** $CD8^+$ memory T cell responses in spleens, DLNs and tumors after re-challenge with CMS5a-NY-ESO-1 and parental CMS5a in mice completely eradicated the initial CMS5a-NY-ESO-1 tumors by treatment with anti-PD-1 mAb with/without ZSTK474 or anti-CD25 mAb. The frequencies of NY-ESO-1 (**D**)- or internal tumor antigen in CMS5a (CMS5a-intAg) (**E**)-specific $CD8^+$ T cells in spleens, DLNs and tumors after 52 days from the primary tumor inoculation. **F.** Effect of anti-CD25 mAb (250 μg/dose; day -1, 150 μg/dose; day 6) on Tregs, NY-ESO-1-specific $CD8^+$ T cells and MPECs. The frequencies of Tregs in $CD4^+$ T cells, NY-ESO-1-specific $CD8^+$ T cells in $CD8^+$ T cells and MPECs in NY-ESO-1-specific $CD8^+$ T cells in spleens, DLNs and tumors 14 days after tumor inoculation. Data in **A** and **D-F** are means ±SE. Data in **B** and **C** show pooled data from two independent experiments. Statistical analyses were performed by Dunnett's test (**A**, **D-F**) and Log rank test (**C**). *, $P < 0.05$; **, $P < 0.01$; ***, $P<0.001$.

Supplemental Figure S1. Antitumor effects and activation of antitumor immunity by ZSTK474 treatment.

A. The cell counts of intra-tumoral Tregs, tumor antigen-specific $CD8^+$ T cells, $CD4^+$ T cells and $CD8^+$ T cells per tumor volume (mm^3) in mice bearing CMS5a-NY-ESO-1 treated with ZSTK474. ZSTK474 was administrated at 30, 100 or 300 mg/kg once a day from day 6 to 13. T cells were collected from tumors at 14 days after tumor inoculation and were subjected to flow cytometry. Data are means ±SD. **B.** Antitumor effects of ZSTK474 in CMS5a-NY-ESO-1 model. ZSTK474 was administrated at 30, 100 or 300 mg/kg once a day from day 6 to 25. The relative changes of tumor volume at day 26 after tumor inoculation are shown. **C.** Phosphorylation status of SLP-76 at Y128 (p-SLP-76) in Tregs, helper $CD4^+$ T cells and $CD8^+$ T cells in DLNs of mice bearing CMS5a-NY-ESO-1 treated with ZSTK474 at 30, 100 or 300 mg/kg once a day from day 6 to 12. DLNs were collected from mice 2 hours after the last administration and T cells in DLNs were subjected to flow cytometry. Representative flow cytometry histograms (upper panels) and summaries of mean fluorescence (MFI) of p-SLP-76 in Tregs, helper $CD4^+$ T cells and $CD8^+$ T cells (lower panels) are shown. **D.** Antitumor effects of ZSTK474 in CMS5a-NY-ESO-1 model in intermittent or continuous dosing protocol. ZSTK474 was administrated at the intermediate dose (100 mg/kg) in each protocol as shown in upper panel. **E.** Changes of body weight (g) of mice treated with ZSTK474 at 100 mg/kg in intermittent or continuous dosing protocol as shown in **D**. **F.** Mice bearing CMS5a-NY-ESO-1 were treated with ZSTK474 in intermittent or continuous dosing protocol as shown in **D**. T cells were collected from tumors at 16 days after tumor inoculation and were subjected to flow cytometry. The frequencies of Tregs in $CD4^+$ T cells in tumors are shown. **G.** Mice bearing CMS5a-NY-ESO-1 were treated with ZSTK474 in continuous dosing protocol. T cells were collected from tumors at 10 days

Isoyama S, et al. J Immunother Cancer 2022; 9:e002279. doi: 10.1136/jitc-2020-002279

and 14 days after tumor inoculation and were subjected to flow cytometry. The frequencies of Tregs in $CD4^+$ T cells in tumors are shown. **H.** Effects of ZSTK474 on PD-1 expression in intra-tumoral $CD8^+$ T cells *in vivo*. Mice bearing CMS5a-NY-ESO-1 were treated with ZSTK474 in continuous dosing protocol. T cells were collected from tumors at 13 days after tumor inoculation and were subjected to flow cytometry. The Mean Fluorescence Intensity (MFI) of PD-1 in $CD8^+$ T cells in tumors are shown. Data are means ±SD. Statistical analyses were performed by Dunnett’s test (**A, B, C, D**) and Student's *t* test (**F, G, H**). *, P < 0.05; **, P < 0.01; ***, P<0.001.

Supplemental Figure S2. Antitumor T cell responses by combination treatment with ZSTK474 and anti-PD-1 mAb.

Representative flow cytometric analysis of **figure 1F** and **G**. $CD4^+CD25^+FOXP3^+$Tregs in spleens (**A**) and in tumors (**B**), and NY-ESO-1-specific $CD8^+$ T cells in tumors (**C**). Mice bearing CMS5a-NY-ESO-1 were treated with or without ZSTK474 and/or anti-PD-1 mAb. T cells were collected from spleens and tumors at 16 days after tumor inoculation and were subjected to flow cytometry. The numbers in the panels indicate the percentage in $CD4^+$ T cells (**A, B**) and in $CD8^+$ T cells (**C**).

Supplemental Figure S3. Intra-tumoral immune cell analyses in the combination treatment with ZSTK474 or Idelalisib and anti-PD-1 mAb.

A-C. Mice bearing CMS5a-NY-ESO-1 were treated with or without ZSTK474 and/or anti-PD-1 mAb in the two different combination protocols (#1 and #2) as shown in **figure 1D**. In #1 combination protocol, ZSTK474 and anti-PD-1 mAb were concurrently administered. In #2 combination protocol, ZSTK474 single treatment was started and then anti-PD-1 mAb plus ZSTK474 combination treatment was followed. T cells were collected from tumors at 16 days after tumor inoculation and were subjected to flow cytometry. **D.** Mice bearing CMS5a-NY-ESO-1 were treated with or without ZSTK474 or Idelalisib and/or anti-PD-1 mAb in #3 combination protocol as shown in **figure 1D**. The changes of the frequencies of Tregs in $CD4^+$ T cells (left) and NY-ESO-1-specific $CD8^+$ T cells in $CD8^+$ T cells (right) in tumors are shown. Data are means ±SD. Statistical analyses were performed by Dunnett's test *, P < 0.05; **, P < 0.01; ***, P<0.001.

Isoyama S, *et al. J Immunother Cancer* 2022; 9:e002279. doi: 10.1136/jitc-2020-002279

Supplemental Figure S4. Antitumor effects by combination treatment with ZSTK474 and anti-PD-1 mAb.

A, B. Antitumor effects of the combination of ZSTK474 and anti-PD-1 mAb in B16F0 tumors. Mice bearing B16F0 melanoma were treated with or without ZSTK474 (once a day from day 10 to 29) and/or anti-PD-1 mAb (day 7, 10, 14 and 17). Tumor growth curves of B16F0 tumors (n = 8 per group) (**A**) and $CD8^+$ T cell/Treg ratio in tumors after 16 days of tumor inoculation (**B**). **C.** Antitumor effects of the combination of high dose ZSTK474 (300 mg/kg) and anti-PD-1 mAb in CMS5a-NY-ESO-1 tumors. Mice bearing CMS5a-NY-ESO-1 were treated with or without ZSTK474 (once a day from day 9 to 28) and/or anti-PD-1 mAb (day 6, 9, 13 and 16). Tumor growth curves of CMS5a-NY-ESO-1 are shown (n = 8 per group). Data are means ±SE. Statistical analyses were performed by Steel's test (**A**) and Dunnett's test (**B, C**). *, $P < 0.05$; **, $P < 0.01$.

Isoyama S, *et al. J Immunother Cancer* 2022; 9:e002279. doi: 10.1136/jitc-2020-002279

Supplemental Figure S5. Inhibition of PI3K signaling by ZSTK474 in *ex vivo* human assays.

A, B. Phosphorylation of S6 protein at S235/236 (p-S6) after stimulation with anti-CD3/anti-CD28 mAb for 30 min with or without ZSTK474 (1 μM). A representative immunoblot analysis (**A**) and average of quantification values of bands (**B**). Data are means ±SE. Statistical analyses were performed by Tukey's test (**B**). *, $P < 0.05$.

Isoyama S, *et al. J Immunother Cancer* 2022; 9:e002279. doi: 10.1136/jitc-2020-002279

Supplemental Figure S6. Increase of memory T cells by combination treatment with ZSTK474 and anti-PD-1 mAb.

A, C, D. Representative flow cytometric analysis of **figure 6A**. $KLRG1^-CD127^+$MPECs in tumors (**A**), $TCF7^+$ T cells in NY-ESO-1-specific $CD8^+$ T cells in tumors (**C**) and $CD62L^+$ T cells in NY-ESO-1-specific $CD8^+$ T cells in DLNs (**D**) are shown. Mice bearing CMS5aY-ESO-1 were treated with or without ZSTK474 and/or anti-PD-1 mAb. T cells were collected from DLNs and tumors 16 days after tumor inoculation and were subjected to flow cytometry. The numbers in the panels indicate the percentage in NY-ESO-1-specific $CD8^+$ T cells (**A, C** and **D**). **B.** Mice bearing CMS5a-NY-ESO-1 were treated with or without ZSTK474 or Idelalisib and/or anti-PD-1 mAb using the optimal protocol. The changes of the frequencies of MPECs in NY-ESO-1-specific $CD8^+$ T cells in tumors are shown. Data are means ±SD. Statistical analyses were performed by Dunnett's test **, $P < 0.01$; ***, $P<0.001$.

Supplemental Figure S7. Effect of anti-CTLA-4 mAb combined with anti-PD-1 mAb on induction of memory T cells.

Effect of anti-CTLA-4 mAb (200 μg/dose; day 9 and 13) on NY-ESO-1-specific $CD8^+$ T cells (**A**) and MPECs (**B**). The frequencies of NY-ESO-1-specific $CD8^+$ T cells in $CD8^+$ T cells and MPECs in NY-ESO-1-specific $CD8^+$ T cells in DLNs and tumors. T cells were collected from DLNs and tumors at 14 days after tumor inoculation and were subjected to flow cytometry. Statistical analyses were performed by Dunnett's test.

Isoyama S, *et al. J Immunother Cancer* 2022; 9:e002279. doi: 10.1136/jitc-2020-002279
