# Supplementary figures and images for: Cancer immunotherapy with PI3K and PD-1 dual-blockade via optimal modulation of T cell activation signal
Source: J Immunother Cancer. 2021 Aug 20;9(8):e002279. doi: 10.1136/jitc-2020-002279 (PMC8395371; doi:10.1136/jitc-2020-002279)

EÉaEö0

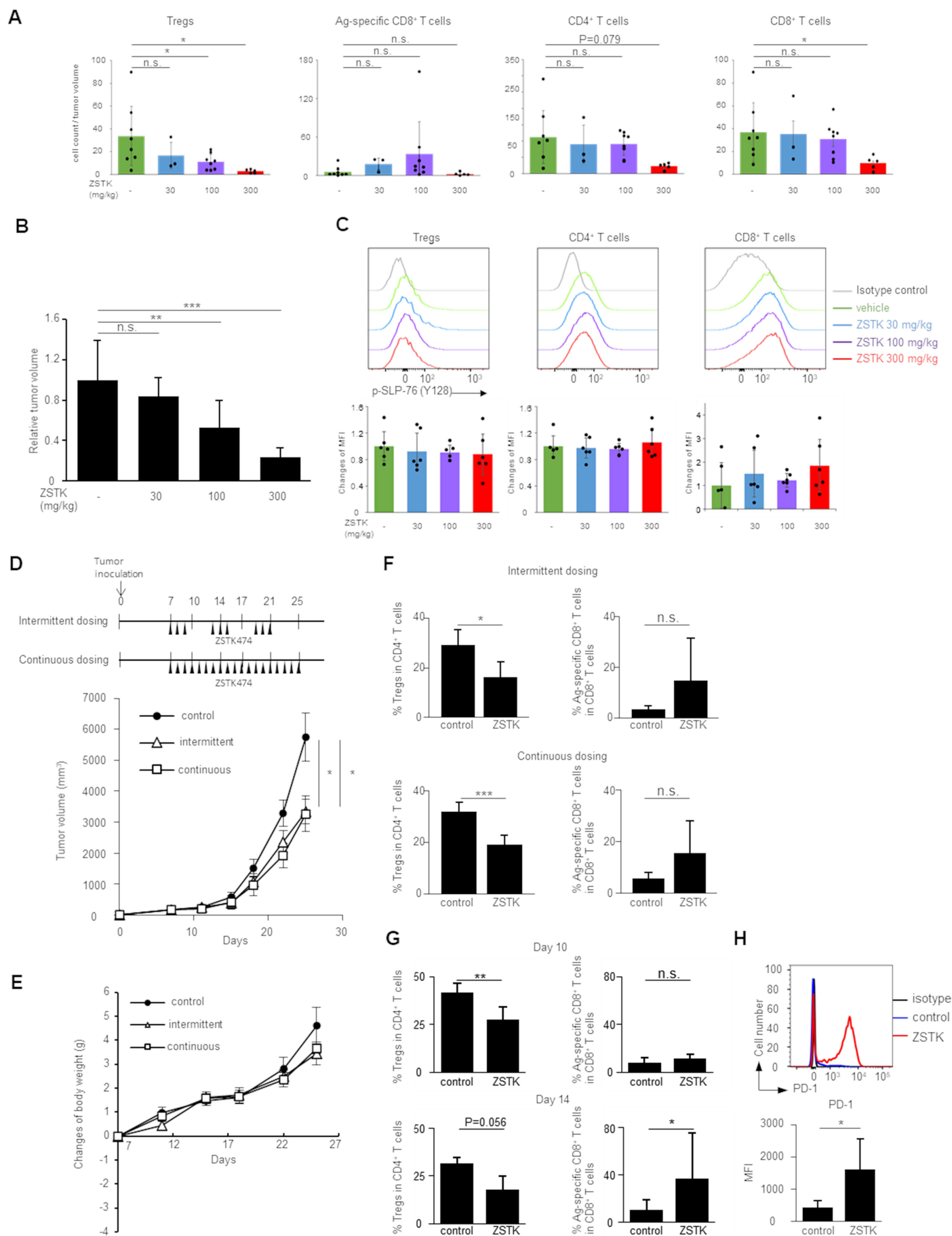

Supplemental figure. S1

Supplement: Supplementary data [file jitc-2020-002279supp003.pdf]

A

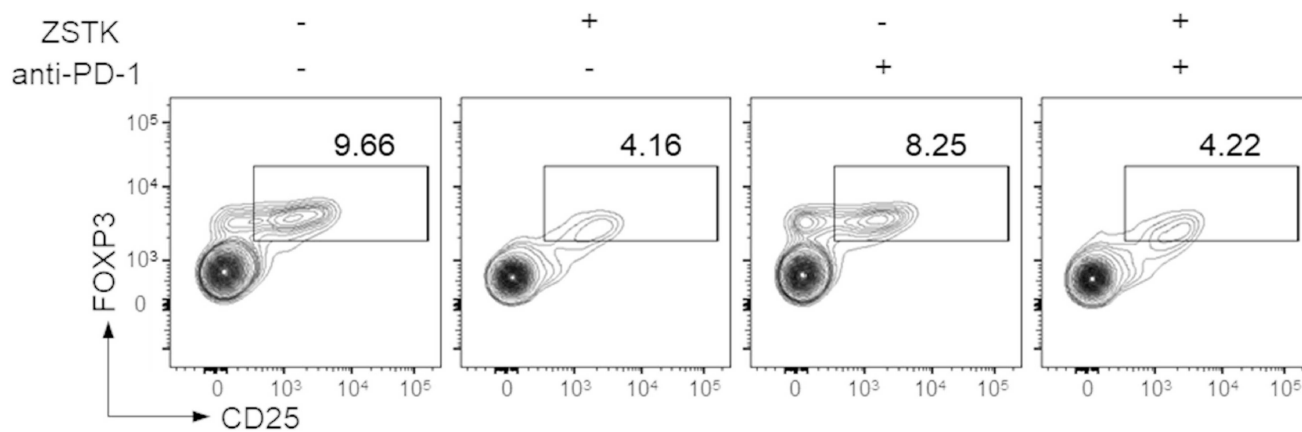

B

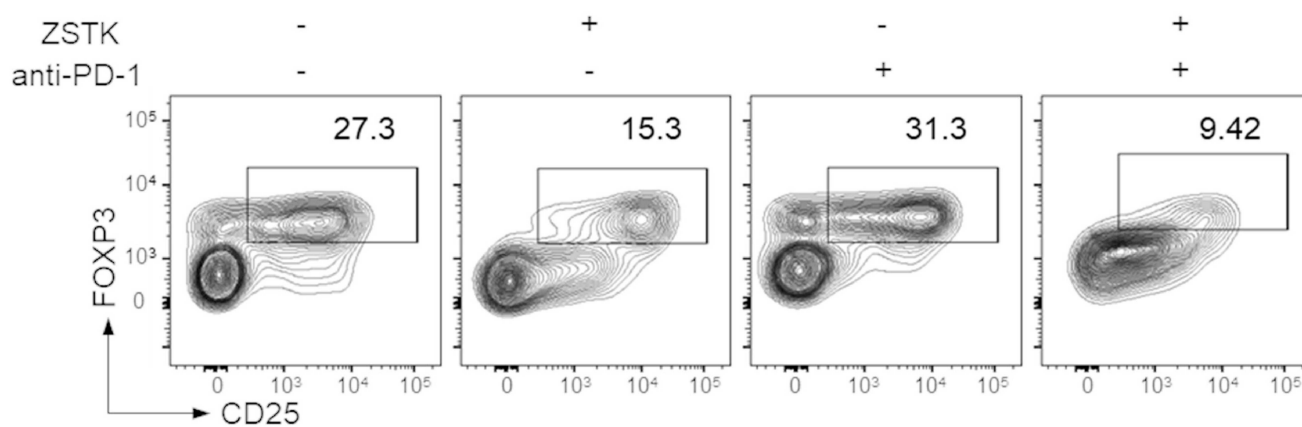

C

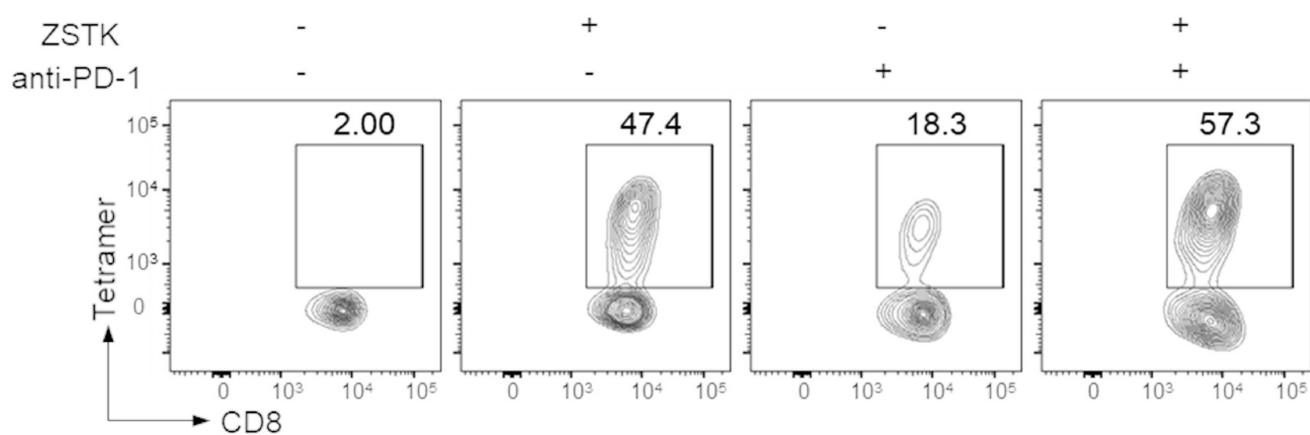

Supplemental figure. S2

Supplement: Supplementary data [file jitc-2020-002279supp004.pdf]

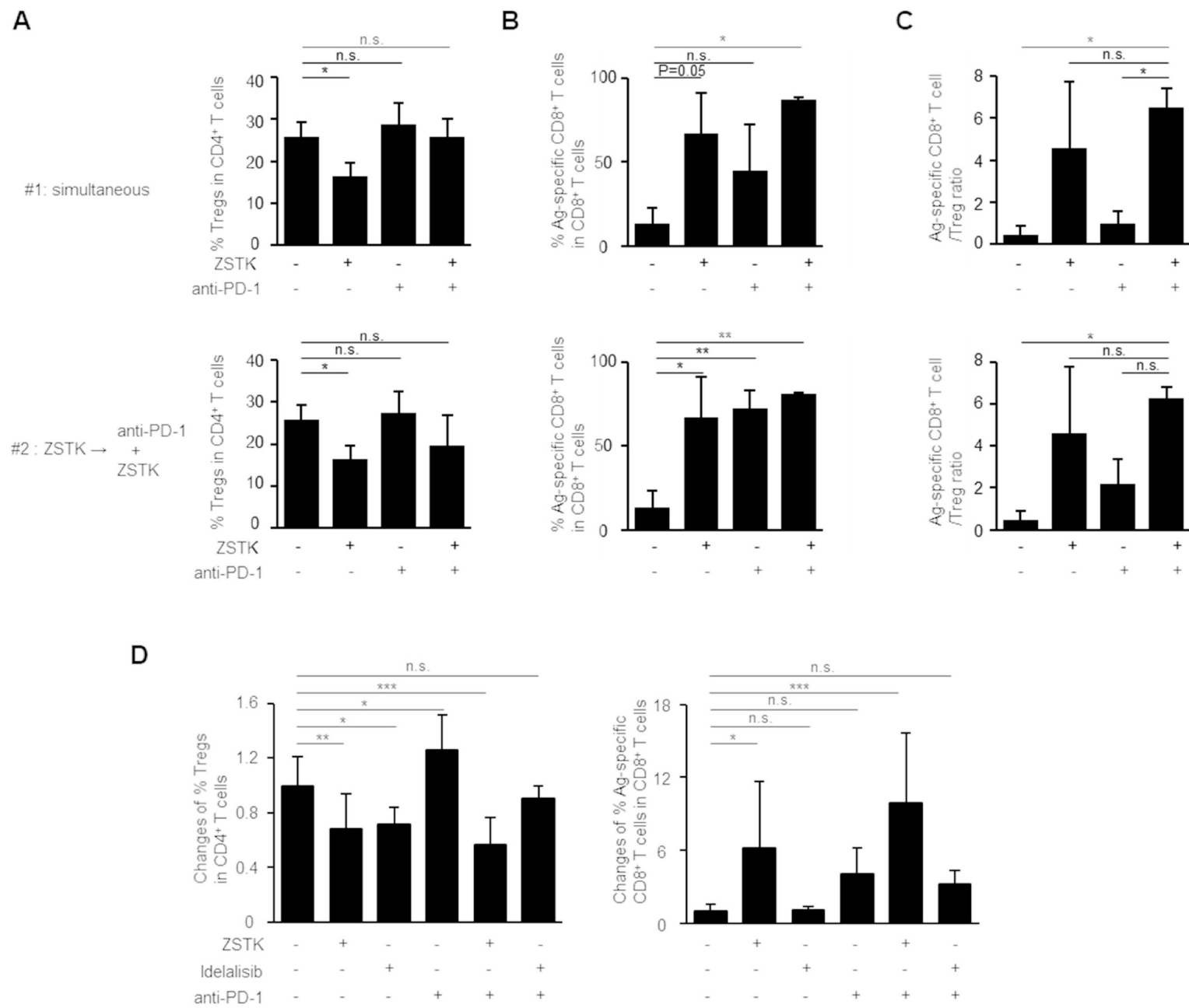

Supplemental figure. S3

Supplement: Supplementary data [file jitc-2020-002279supp005.pdf]

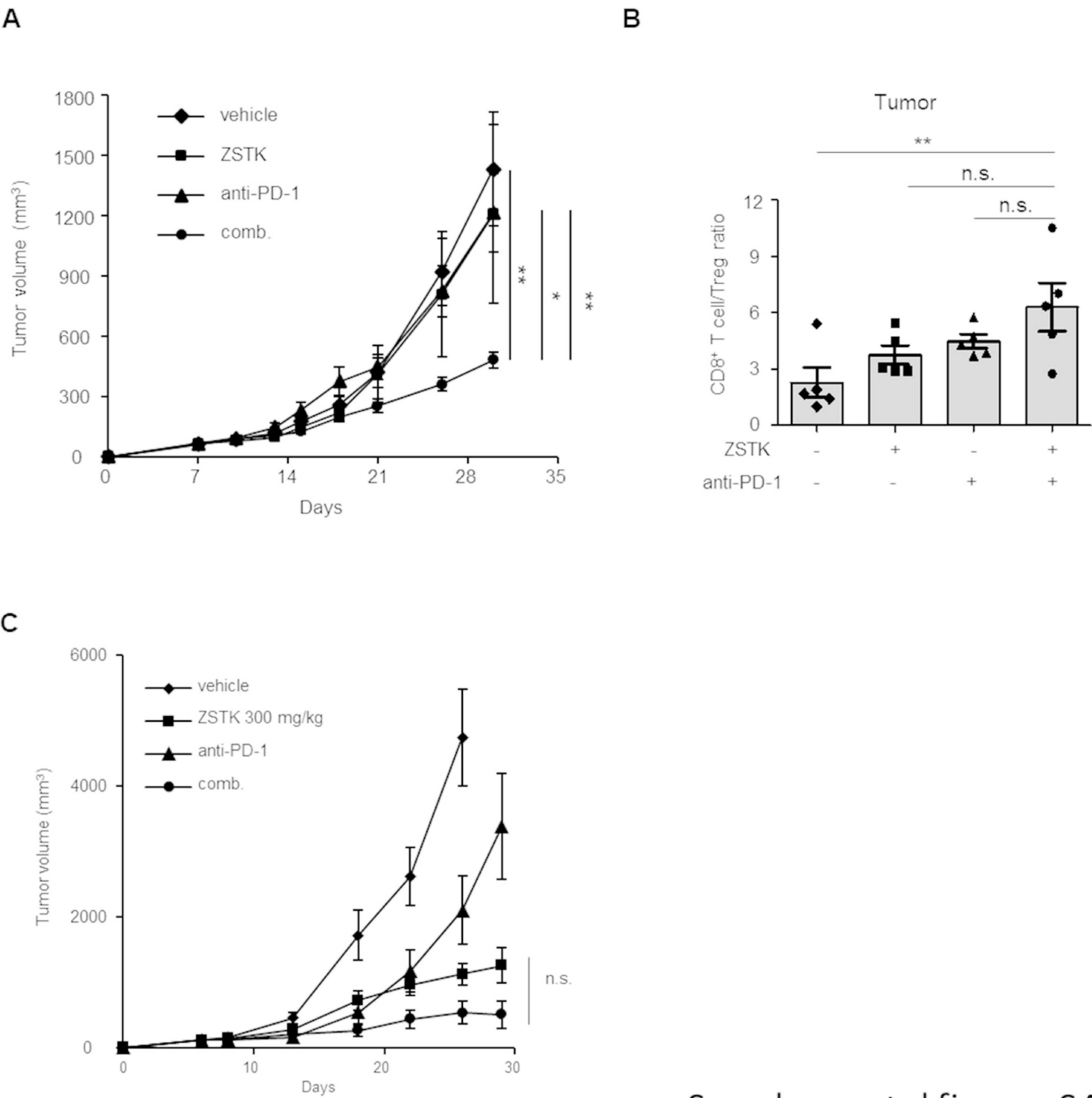

Supplemental figure. S4

Supplement: Supplementary data [file jitc-2020-002279supp006.pdf]

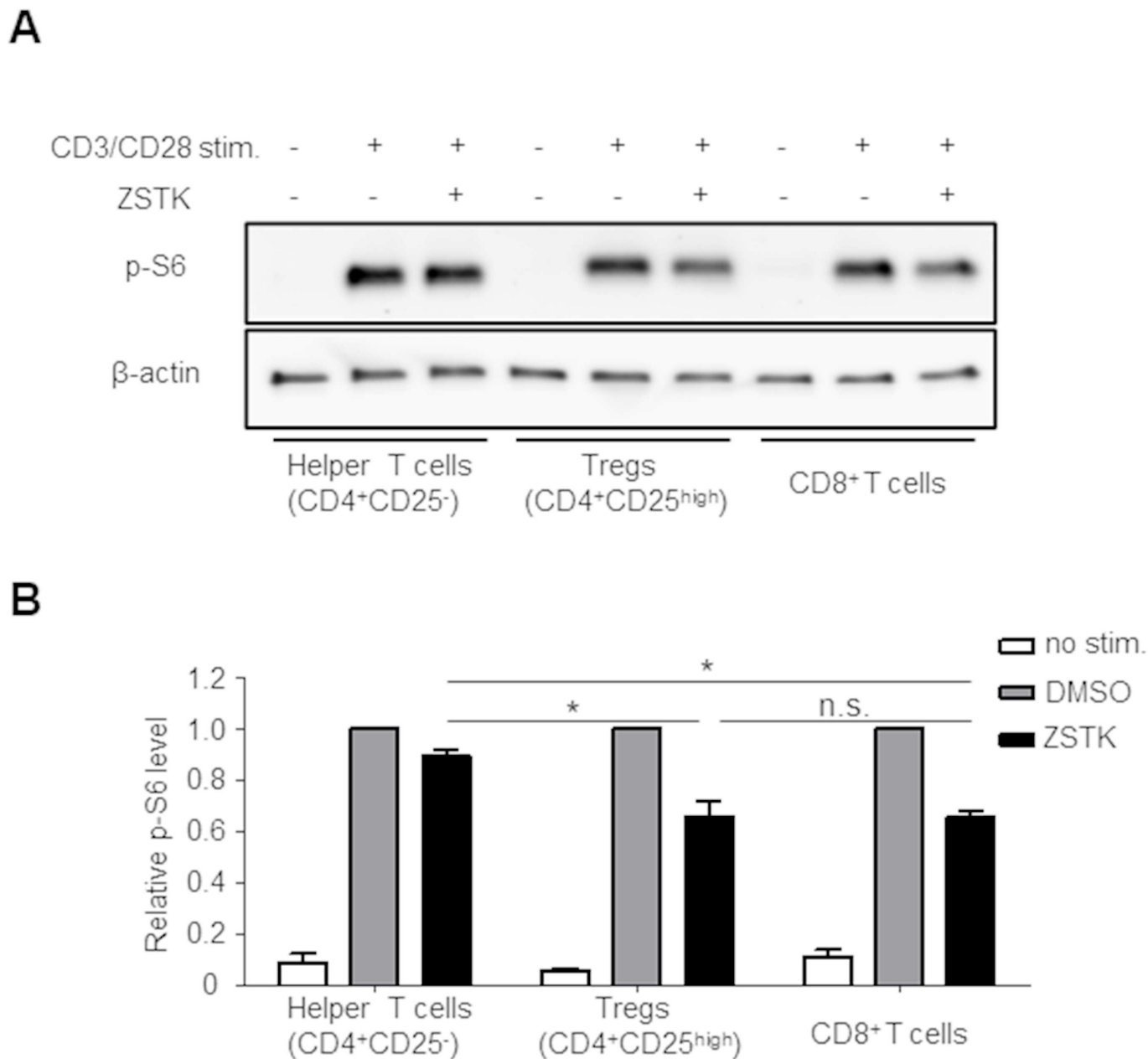

Supplementary figure. S5

Supplement: Supplementary data [file jitc-2020-002279supp007.pdf]

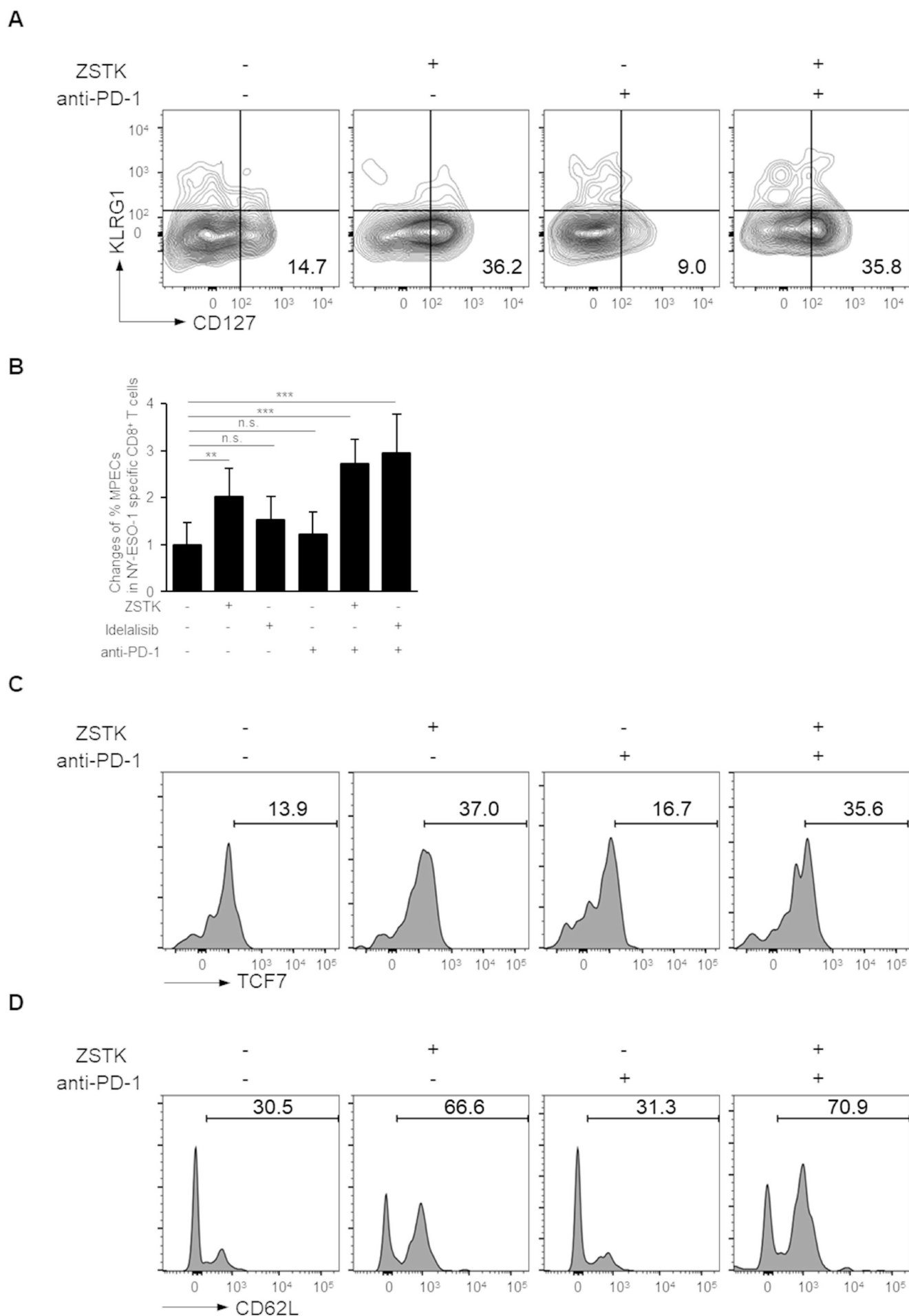

Supplementary figure. S6

Supplement: Supplementary data [file jitc-2020-002279supp008.pdf]

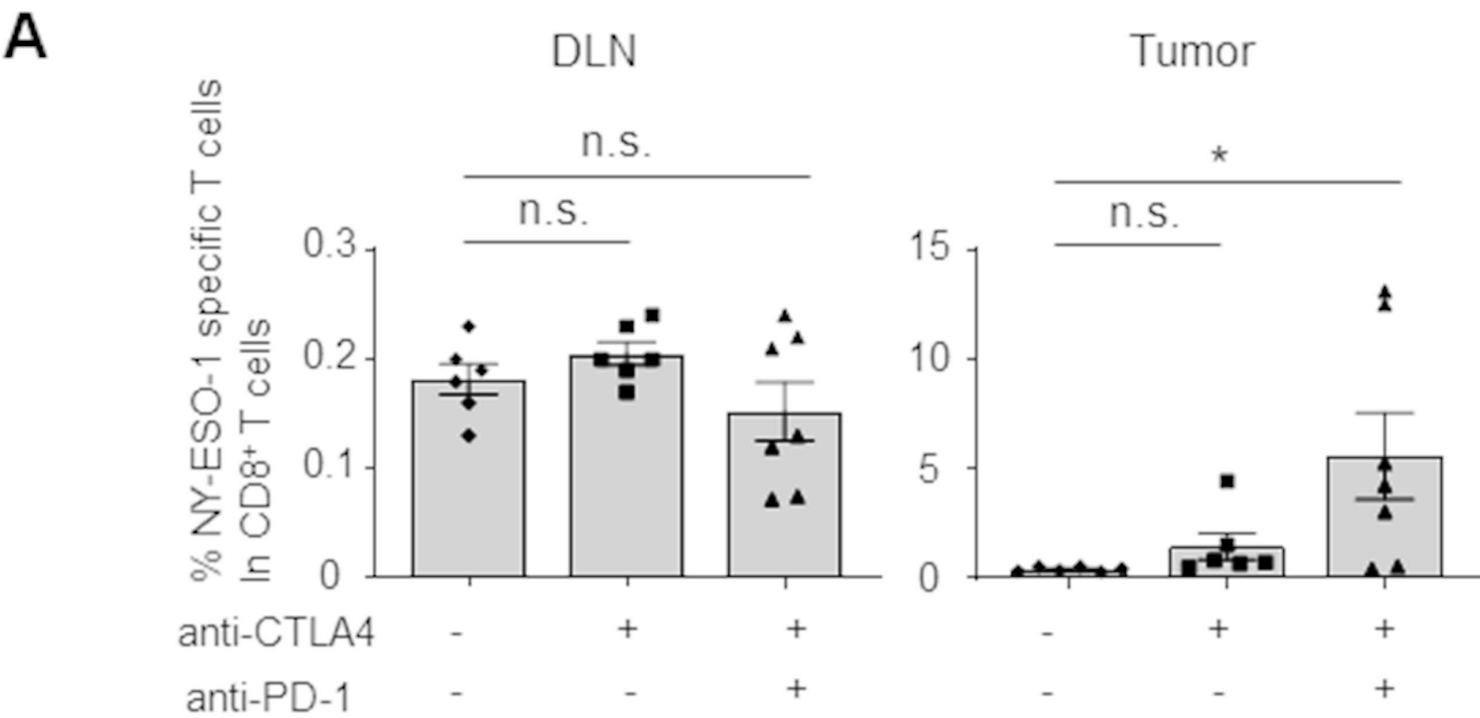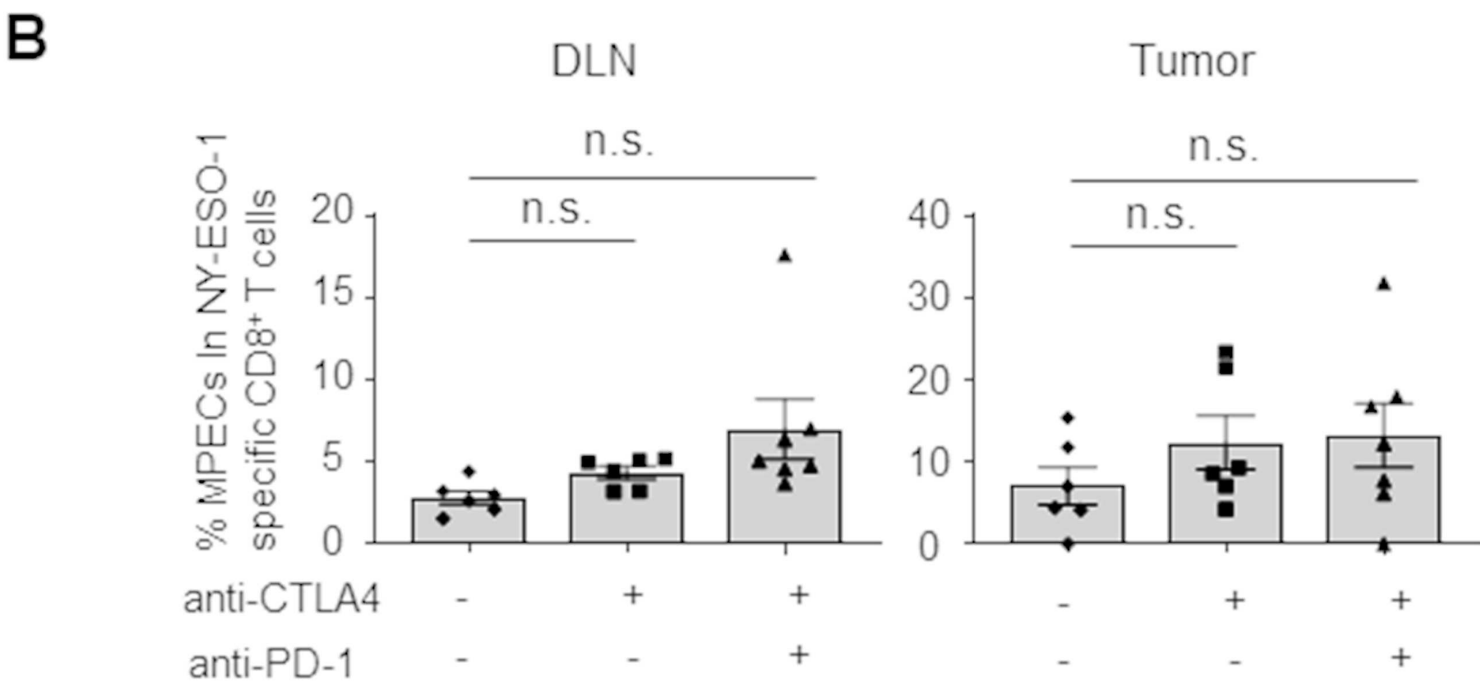

Supplemental figure. S7

Supplement: Supplementary data [file jitc-2020-002279supp009.pdf]
